# LE-PWDNet: a lightweight and enhanced detection framework based on DEIM for early-stage pine wilt disease

**DOI:** 10.3389/fpls.2025.1701009

**Published:** 2025-12-15

**Authors:** Yujia Shen, Fang Wang, Jingjing Qian, Haifeng Lin

**Affiliations:** 1 College of Forestry, Nanjing Forestry University, Nanjing, China; 2 College of Electronic Engineering, Nanjing Xiaozhuang University, Nanjing, China; 3 College of Engineering, Mcmaster University, Hamilton, ON, Canada

**Keywords:** pine wilt disease, early-stage object detection, lightweight detection model, UAV RGB imagery, DEIM

## Abstract

Pine wilt disease (PWD), characterized by rapid transmission and high pathogenicity, causes severe ecological and economic damage worldwide. Early detection is critical for curbing its spread, yet the concealed symptoms and minute lesions make it difficult for existing models to balance high accuracy with lightweight efficiency in complex forest environments. To address these challenges, this study proposes a lightweight detection model named LE-PWDNet. A total of 41,568 high-resolution UAV images were collected from diverse field scenarios to construct a standardized dataset covering four infection stages, providing comprehensive support for model training and evaluation. The model is built upon the DEIM training paradigm to enhance the utilization of positive samples for small-target detection. To strengthen multi-scale texture modeling of early lesions, a Wavelet Detail Attention Convolution (WDAConv) is designed. A ConvFFN module is introduced to mitigate the attenuation of high-frequency details, thereby improving robustness under complex backgrounds. A CGAFusion module is developed to reduce false positives caused by background noise. Furthermore, an Edge-Dilated Sampling-Point Generator (DySample-E) is incorporated to dynamically adjust the upsampling process, enhancing the ability to capture early micro-lesions. Experimental results demonstrate that, with only 5.64M parameters and approximately 7 GFLOPs, LE-PWDNet achieves an AP_50_ of 83.8% for early-stage lesion detection and an overall AP_50_ of 90.2%, outperforming existing mainstream models. This study provides a feasible solution for building intelligent and low-cost early-warning systems for forest diseases and highlights the broad application potential of the proposed framework in forestry and other ecological monitoring scenarios.

## 1 Introduction

Pine Wilt Disease (PWD), also known as pine wood nematode disease, is a devastating forest disease caused by Bursaphelenchus xylophilus (Li et al., 2022). The pathogen originated in North America and has spread widely to East Asia in recent years. It spreads rapidly and has strong pathogenicity. If it is not discovered in time, it will cause death of large areas of pine trees and cause serious ecological and economic losses (Back, 2020). In the 40 years since its invasion of China, the disease has caused disastrous economic and ecological losses, with the combined economic and ecosystem service value losses estimated at approximately US $7.4 billion in 2020 (Liu et al., 2023a). Therefore, early detection and accurate location of infected pine trees are very important to control the spread of epidemic diseases, which not only helps to protect forest resources and maintain ecosystem stability, but also has important value for promoting sustainable social and economic development.

Traditional detection methods primarily rely on manual on-site inspections. However, due to environmental factors such as rugged forest roads and complex mountainous terrain, conducting comprehensive inspections is extremely challenging. Manual patrols not only involve high labor intensity and low work efficiency but also fail to meet the requirements for efficient and precise detection and epidemic prevention in large-scale forest areas. Therefore, combining drone imagery with deep learning to achieve automatic identification has become a current research focus. Drones offer advantages such as high efficiency, low cost, and wide coverage, enabling the rapid acquisition of high-resolution images over large areas, thereby providing strong support for early detection. Additionally, the rapid development of computer vision and deep learning has also provided robust support for disease identification.

Convolutional neural network (CNN), as a representative algorithm of deep learning, shows excellent performance in pest detection tasks. In 2020, Deng et al. (2020) realized high-precision disease tree identification based on optimized Faster R-CNN. Yu et al. (2021) found another way to enhance the spatial-spectral modeling ability of hyperspectral images by constructing a three-dimensional convolutional neural network (3D-CNN) integrating residual blocks. In 2023, Wu and Jiang (2023) proposed to introduce improved Mask R-CNN into GN and WS normalization, and fuse ConvNeXt structure to improve the accuracy of disease extraction. Meanwhile, the advent of the YOLO (You Only Look Once) family has shifted object detection toward single-stage architectures, which provide higher efficiency than traditional two-stage models and has achieved significant success in image recognition. In 2024, Ye et al. (2024) introduced the SOCA attention mechanism and the WBF strategy, combined with StrongSORT (Du et al., 2023), to enhance the performance of YOLOv5 in object tracking and counting in complex forest environments; in the same year, Du et al. (2024) focused on the development of lightweight detection model and proposed lightweight YOLO model integrating ShuffleNetV2, SimAM and ASFF, realizing high-precision real-time detection. Although CNN is good at local feature extraction, it has limitations in modeling long distance dependence and global semantics, especially in the face of early subtle lesions, complex backgrounds and occlusion interference (Li et al., 2024). YOLO has good real-time performance, but it is not adaptive enough in complex scenes, and highly dependent on non-maximum suppression (NMS), which not only increases reasoning delay, but also needs to manually adjust hyperparameters to balance accuracy and efficiency, thus limiting its application in intelligent forest disease detection.

Unlike CNNs, attention and Transformer architectures emphasize modeling spectral–spatial correlations from a more global perspective and have therefore been increasingly introduced into remote sensing and hyperspectral image (HSI) analysis. Liu et al. (2022a) proposed the Central Attention Network (CAN), which employs a center-pixel–oriented attention mechanism that selectively retains only spectrally similar neighboring pixels, yielding cleaner spectral and spatial representations in complex backgrounds. They subsequently introduced the Multiarea Target Attention (MATA) model (Liu et al., 2023b), where multiscale target-region attention and class-wise weighted decision fusion enhance the representation of discriminative spectral–spatial regions at different scales and positions, thereby improving the separability of small lesions under complex canopy conditions. However, such pixel-wise processing still restricts the effective use of spatial information. To alleviate this, Liu et al. (2024a) reformulated HSI classification from a semantic-segmentation perspective and proposed SegHSI, which integrates cluster attention, a spatial-aware feedforward network, and student-teacher consistency learning to perform efficient end-to-end segmentation of entire HSIs under limited model. Furthermore, in the specific context of pine wilt disease, Liu et al. (2024b) designed Clusterformer, a Transformer-based segmentation framework that leverages cluster-level feature representations and layer-wise propagation to suppress background interference and strengthen the representation of diseased pine trees, achieving substantially higher segmentation accuracy than mainstream segmentation methods on two PWD datasets. However, although these methods are advantageous for the spectral–spatial characterization of disease lesions, they rely on hyperspectral data with high acquisition cost and operational complexity, together with structurally complex attention and Transformer networks, which makes them difficult to be widely applied in UAV-based pine wilt disease monitoring scenarios that require high-frequency inspection and large-area coverage.

To solve these problems, DETR (Carion et al., 2020) (Detection Transformer) is proposed as a novel end-to-end detection model. For the first time, it regards the target detection task as a set prediction problem and combines Transformer and Hungarian matching algorithm to construct an end-to-end detection framework, which significantly simplifies the candidate box generation and post-processing flow in traditional methods. Compared to CNN-based single-stage detectors (e.g., YOLO series) and two-stage detectors (e.g., Faster R-CNN), DETR adopts a fully end-to-end trainable architecture without design constraints such as NMS post-processing, prior knowledge, or anchor boxes, thus significantly simplifying the detection process. However, DETR has some problems such as slow training convergence, weak detection ability of small targets, insufficient local feature expression and low reasoning efficiency, which limit its application in practical scenes (Shehzadi et al., 2023). As a result, research on improvements around DETR continues to advance. To this end, RT-DETR (Zhao et al., 2024) optimizes the original architecture, significantly improves the feature fusion efficiency and query initialization quality by introducing hybrid encoder and IoU sensing mechanism, and achieves end-to-end real-time detection performance close to YOLO for the first time. Although RT-DETR has excellent performance in speed and accuracy, it is still difficult to accurately describe the shape and boundary of complex targets by using traditional four-parameter regression method.

In contrast, DEIM (Huang et al., 2025) has demonstrated notable advantages in detecting early-stage PWD, which is typically characterized by small target size, subtle visual features, dense spatial distribution, and the need for real-time response. By introducing a dense one-to-one matching strategy (Dense O2O) and matchability-aware loss (MAL), DEIM significantly increases the number of positive samples and optimizes the training signal, thereby accelerating convergence and improving the stability of small-target detection. Compared with traditional CNN-based methods, DEIM achieves superior detection accuracy within shorter training times. Relative to advanced models such as RT-DETR and D-FINE (Peng et al., 2024), DEIM offers better scalability and application potential while maintaining low latency, balancing detection accuracy with training efficiency. Nevertheless, despite its promising real-time performance, DEIM’s exploration in the early detection of PWD remains limited, and further research is required to extend its applicability. Moreover, most existing studies evaluate detection models on a single-site dataset, which may not adequately reflect cross-regional generalization. To address this gap, we additionally conducted a zero-shot evaluation on a UAV-RGB dataset collected from a geographically distinct PWD outbreak region, enabling direct assessment of the proposed method’s adaptability to unseen forest environments.

In response to the challenges posed by early-stage PWD detection, we propose a lightweight detection model, LE-PWDNet, built upon the DEIM training paradigm. This framework achieves fast and accurate disease identification while reducing computational complexity without sacrificing detection accuracy. The main contributions of this work are summarized as follows:

1. Based on UAV-based data acquisition, a high-quality PWD dataset was established, covering four infection stages—early, middle, late, and dead.
2. We introduce the lightweight LE-PWDNet architecture, which incorporates: (a) a Wavelet Detail Attention Convolution (WDAConv) that integrates wavelet transform, Detail Enhanced Convolution (DEConv), and an Adaptive Parallel Attention Mechanism (APAM) to efficiently capture texture, edge, and multi-scale structural cues; (b) a multi-branch APAM to fuse channel-, spatial-, and original feature-level information for improved perception and localization of weak early-stage lesions; (c) a ConvFFN module to enhance the Transformer’s capacity for high-frequency detail modeling, thereby improving responsiveness to subtle chlorotic symptoms in aerial imagery; (d) a CGAFusion module that constructs a cross-layer fusion pathway via channel–space–pixel-level attention to ensure semantic consistency; and (e) a DySample-E learnable upsampling module that combines dilated convolution and edge-residual guidance to strengthen structural continuity and reduce missed detections or boundary misclassifications of small targets.
3. Comprehensive evaluation on the self-constructed early PWD dataset, supported by extensive ablation studies, confirms the contribution of each module, while comparative experiments verify the overall superiority of the proposed framework.
4. This study provides a reliable pathway for early and accurate PWD detection, enhancing the management efficiency and operational quality of agroforestry systems. By enabling timely monitoring and intervention, the proposed approach can curb disease spread during critical phases, safeguard the ecological security of pine forests, and promote sustainable and efficient development of the forestry industry.

## 2 Materials and methods

### 2.1 Study area

**Figure 1:**
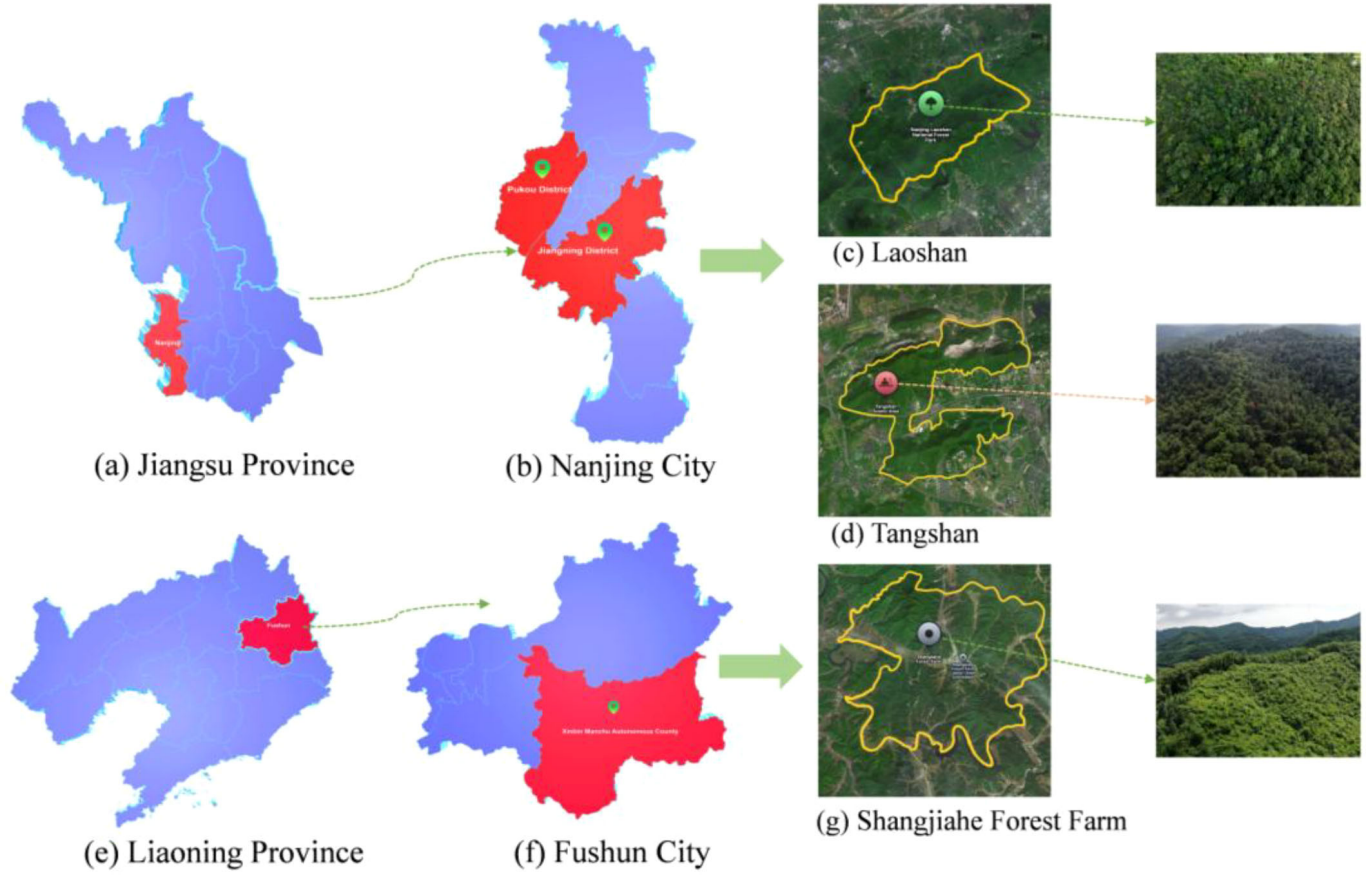
Study area of this paper. **(a)** Jiangsu Province. **(b)** Nanjing City. **(c)** Laoshan. **(d)** Tangshan. **(e)** Liaoning Province. **(f)** Fushun City. **(g)** Shangjiahe Forest Farm.

The study areas of this paper are located in Laoshan National Forest Park, Pukou District, Nanjing City, Jiangsu Province, China (N30°39′00″ to N30°41′00″, E118°29′00″ to E118°31′00″),Tangshan Scenic Area, Jiangning District, Nanjing City, Jiangsu Province (N32°00′20″ to N32°06′10″, E118°57′20″ to E119°05′40″) and the Shangjiahe Forest Area of Xinbin Manchu Autonomous County in Fushun City, Liaoning Province. The geographical locations are shown in **Figure 1**. Laoshan National Forest Park is densely forested, with a forest coverage rate of more than 95%, of which 94,000 mu of forest land is dominated by deciduous forest, evergreen broad-leaved forest, and coniferous forest, including Pinus massoniana, Pinus elliottii, Pinus taeda, fir, Taxus chinensis, Platycladus orientalis, and Taxus chinensis. Tangshan Scenic Area contains approximately 150,000 mu of mountainous forest with dense vegetation, and its dominant tree species include Pinus koraiensis, Cedrus deodara, and Metasequoia glyptostroboides. In contrast, the Shangjiahe Forest Area represents a typical temperate mountain forest ecosystem in Northeast China, dominated by natural needle–broadleaf mixed forests. Representative coniferous species include Pinus koraiensis, Pinus sylvestris var. mongolica, and Picea jezoensis. The region experiences a markedly colder climate with a shorter growing season. Notably, the three areas differ considerably in terrain, forest structure, and vegetation composition, providing diverse ecological scenarios for collecting PWD-infected wood image data and enhancing the representativeness and applicability of the constructed dataset.

### 2.2 Data acquisition and preprocessing

#### 2.2.1 UAV-based image acquisition of pine wilt disease

In this study, *in-situ* aerial imagery was acquired using the DJI Mavic 3 UAV. The UAV weighs 951 grams, with a maximum horizontal flight speed of 15 m/s and an endurance of up to 43 minutes. This configuration allows wide forest coverage and supports accurate monitoring of PWD and associated forest pests. A 4/3-inch CMOS Hasselblad camera is integrated into the Mavic 3, offering 20 MP effective resolution and supporting RAW-format RGB image acquisition. This setup enables high-resolution detection of early pest symptoms, such as needle yellowing and canopy thinning. The integrated DJI O3+ image transmission system delivers real-time high-definition imagery with a range of up to 15 km and latency below 120 ms, ensuring temporal precision and stability in data acquisition. In addition, the UAV’s omnidirectional obstacle avoidance and autonomous flight planning enable safe operation in complex mountainous and forested environments. Collectively, these capabilities establish the Mavic 3 UAV as an effective platform for large-scale dynamic monitoring of PWD.

**Figure 2:**
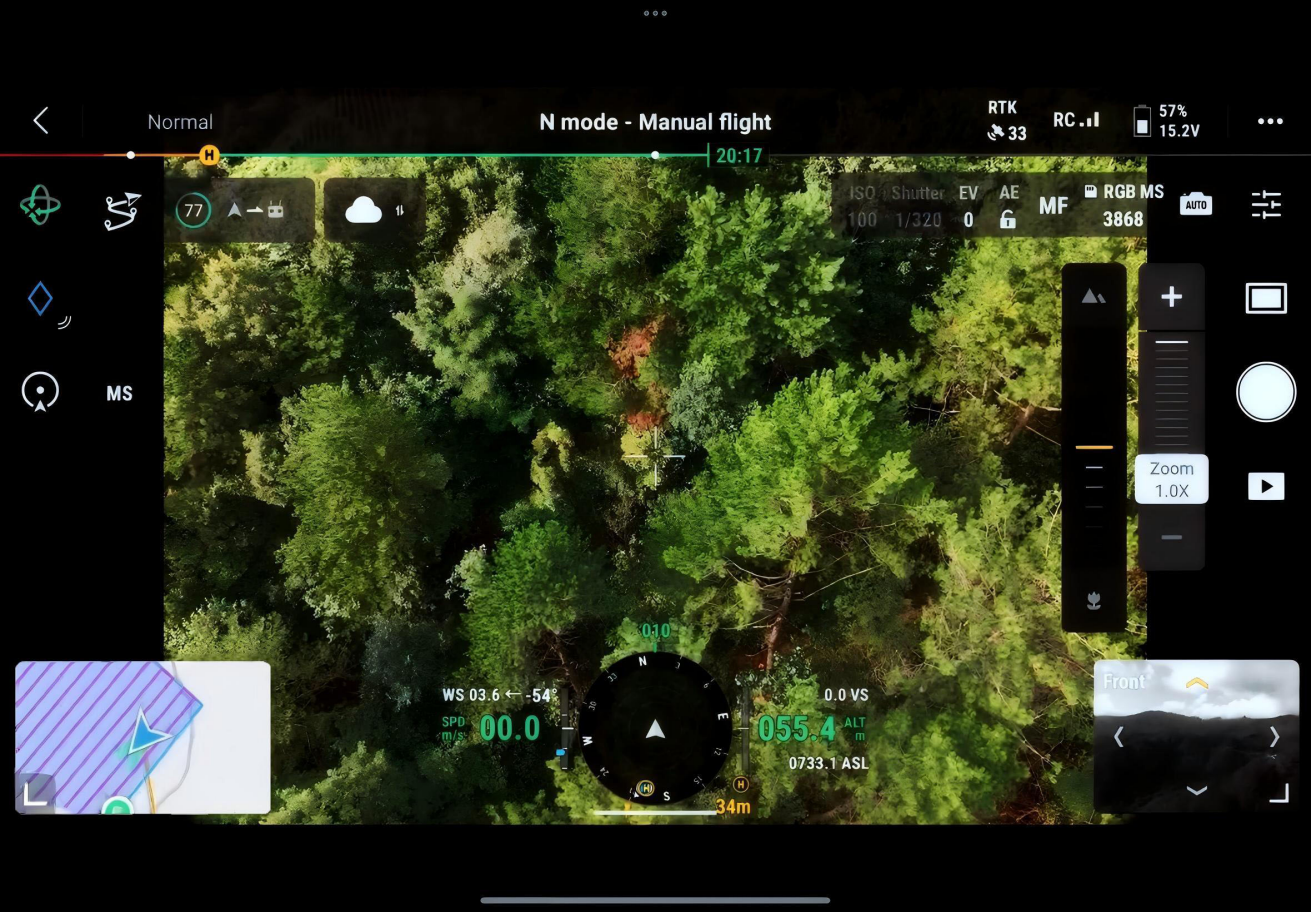
UAV Remote Control Interface.

Aerial image data were collected using the DJI Mavic 3 UAV. Three acquisition campaigns were conducted in March, June, and September 2024. During image acquisition, the UAV’s flight area was manually delineated, and images were captured at equal intervals to ensure uniform coverage and data completeness. To accommodate the complex topography and variable vegetation density, planar flight route planning was implemented to maximize coverage and minimize flight path overlap. The real-time flight status is illustrated in the accompanying **Figure 2**.

**Figure 3:**
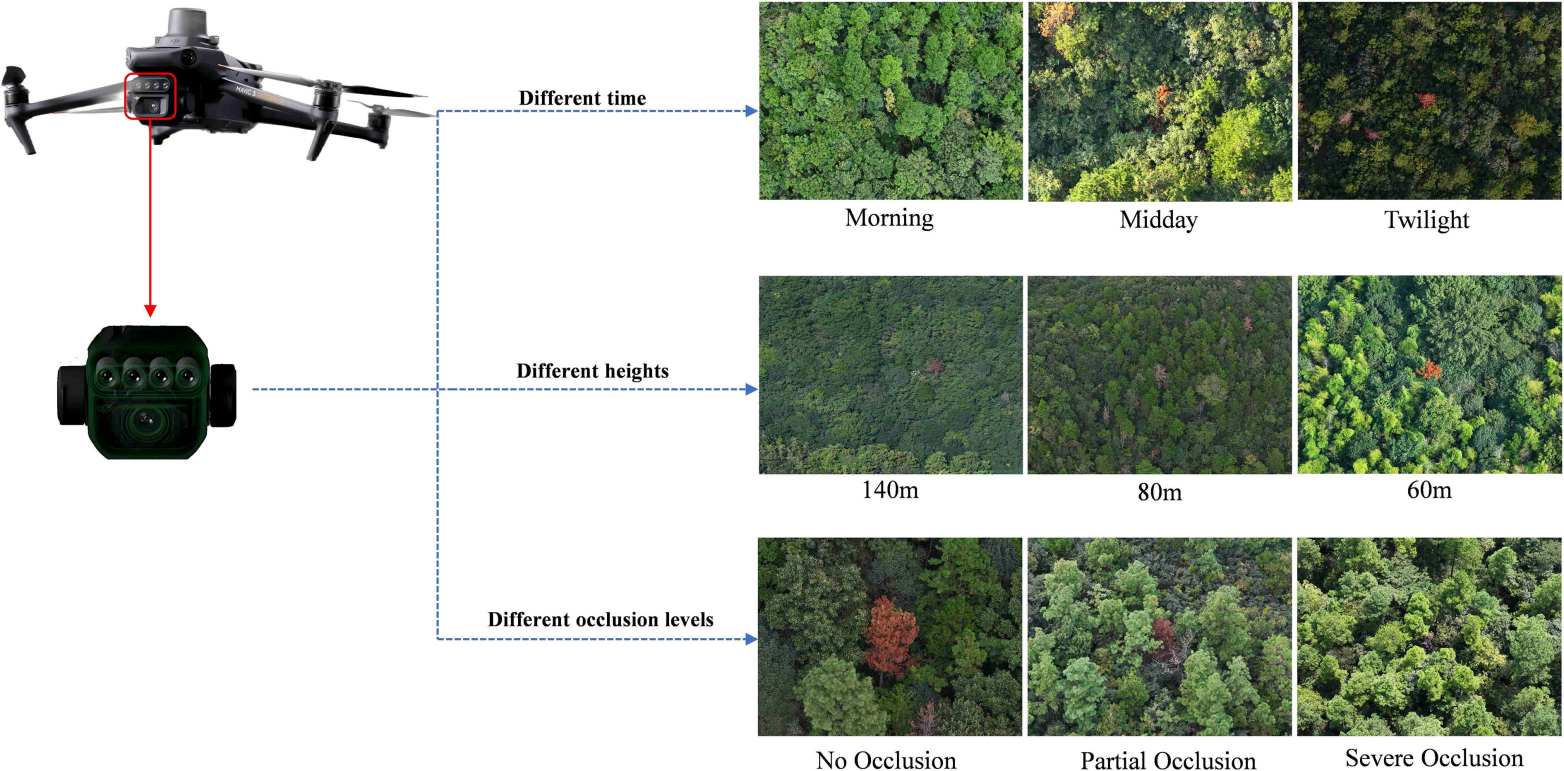
UAV images acquisition.

To enrich the diversity and comprehensiveness of the PWD dataset, PWD images were collected at different time periods (morning, noon and dusk), at different shooting heights (140 m, 80 m and 60 m above ground) and at different infection levels to ensure that the dataset contained PWD information under various environmental conditions. After more than 36 flights, 41568 high-resolution images were collected. An example of partial image data is shown in **Figure 3**.

#### 2.2.2 UAV-based image preprocessing

According to the symptoms of PWD, targets with sizes between 4×4 pixels and 8×8 pixels are generally classified as small targets. Because of their small pixel area in the image, these targets are easily interfered by background clutter and other objects, which leads to the accuracy degradation of existing detection algorithms. In this study, PWD targets were mostly characterized as small targets in the high-resolution remote sensing images obtained from high altitude by unmanned aerial vehicle (UAV), that is, the target area occupied a small pixel area in the whole image and was easily confused with the complex forest background.

**Figure 4:**
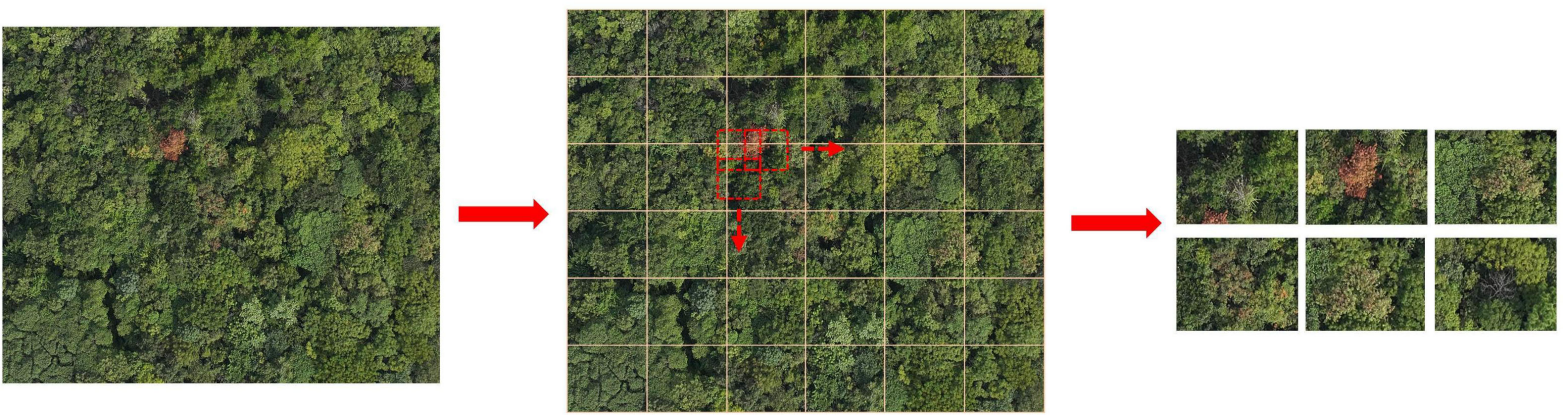
Sliding window slicing process.

To enhance the detection accuracy of PWD of small targets and enhance the diversity of sample data sets, a slicing method based on sliding window is introduced in this paper. By dividing the original large image into several local regions, this method reduces the attention range of the model and helps to enhance the saliency of small targets in the background. **Figure 4** shows the sliding window slicing process. In this study, the original image is divided into 36 sub-regions evenly, and the overlap rate between regions is set to 10% to reduce the probability of truncation when the target is located in the edge region.

Within each local area, a sliding window with a fixed size of 200×200 pixels is applied for slicing, which allows for controllability of computational costs while maintaining local detail. In order to enhance context information sharing and feature continuity between windows, the sliding step size is set to 180 pixels, so that there is a reasonable overlap area between adjacent slices, thus improving the sensitivity and stability of the model to the target edge area in the detection phase.

#### 2.2.3 Dataset construction

**Figure 5:**
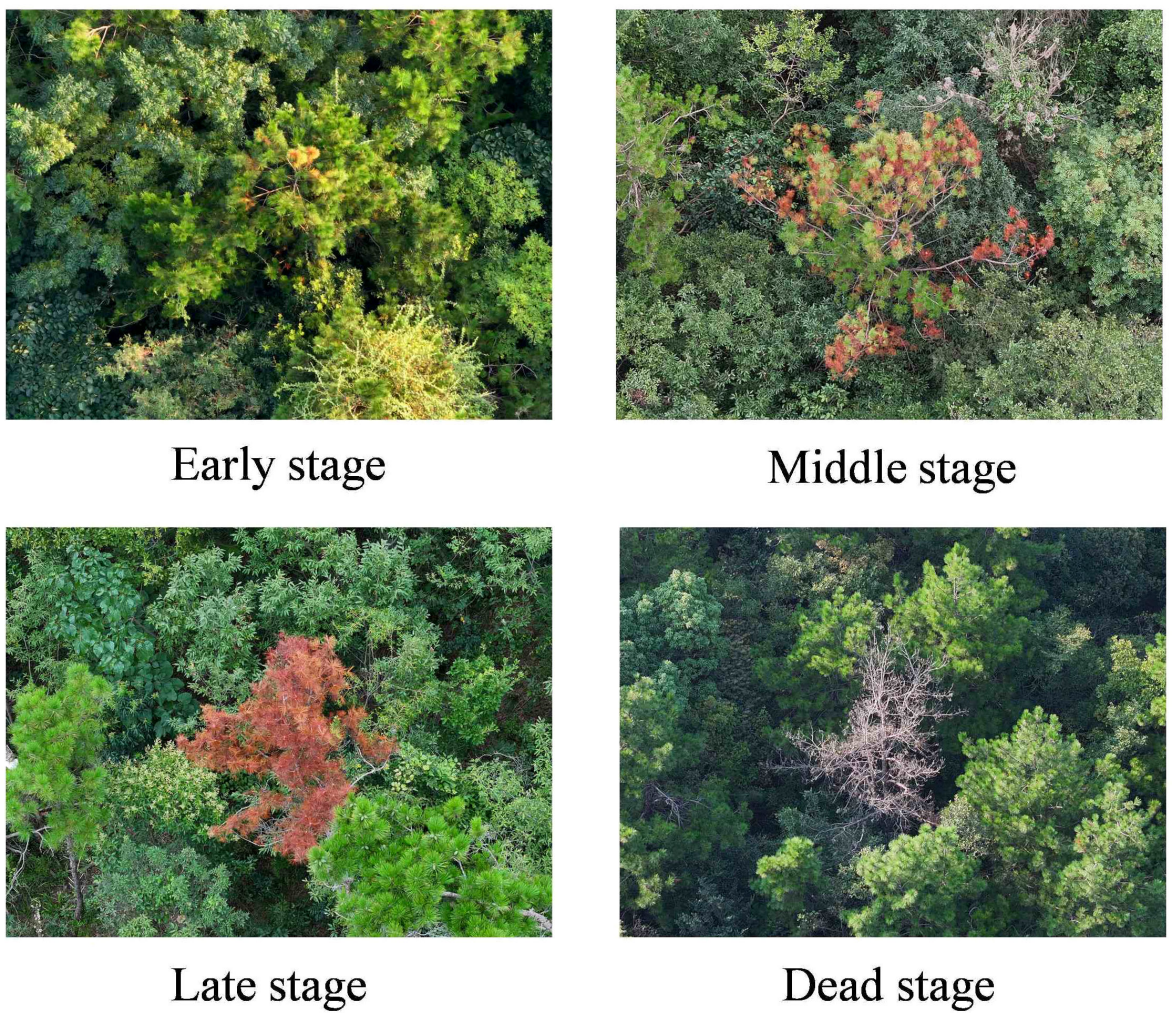
Images of diseased pine trees at different stages.

Based on the clinical manifestations and visual characteristics of PWD, the infection process was categorized into four distinct stages, which are early, middle, late, and dead tree, as illustrated in **Figure 5**. In the early stage, needles typically exhibit star-shaped yellowing, initially occurring at the crown apex or in localized clusters and gradually spreading outward. In the middle stage, needles wither extensively, with infected areas turning yellow or grayish yellow. In the late stage, needles display widespread yellowing and wilting, accompanied by a decline in tree vitality; trunks lose their green hue and turn dark brown. In the dead tree stage, needles and branches are almost entirely shed, with evident signs of decay; trunks appear gray or dark brown.

The primary dataset was collected from Laoshan National Forest Park, Pukou District, Nanjing, China, using a UAV-based imaging platform. High-quality images were rigorously screened and manually annotated for early infection regions using the LabelImg tool. This dataset comprises 36,845 high-resolution PWD images, which were split into training and validation sets in an 8:2 ratio, containing 29,476 and 7,369 samples, respectively, and was used exclusively for model training and in-domain performance evaluation.

To assess cross-regional generalization, two independent datasets were additionally collected from Tangshan Scenic Area, Jiangning District, Nanjing, and the Shangjiahe Forest Area in Fushun City, both of which differ markedly from Laoshan in terrain, forest structure, and dominant tree species. Following the same annotation protocol and preserving the original resolution, these datasets contain 2560 and 3920 images exhibiting early-stage PWD symptoms. They were excluded from all training and hyperparameter tuning and were used solely for zero-shot evaluation, enabling a direct assessment of the proposed model’s adaptability to previously unseen ecological conditions.

### 2.3 LE-PWDNet

The task of early PWD image detection faces multiple challenges, including extremely small target size, weak disease spot characteristics, dense distribution and easy occlusion interference, especially in complex forest background, which easily leads to serious imbalance of positive and negative samples, thus affecting the modeling ability and training stability of the model for early weak symptoms. In order to enhance the perception ability of the detection model for early lesions and improve the effectiveness of the training signal, DEIM (DETR with Improved Matching for Fast Convergence) is introduced as the training paradigm. This method significantly increases the number of positive samples through dense one-to-one matching, strengthens the learning frequency of small targets, introduces matchability-Aware Loss, adaptively allocates optimization weights according to matching quality, effectively suppresses interference caused by low-quality supervision, and improves the overall training robustness and feature expression ability. In view of the requirement of lightweight model, DEIM-D-Fine-N, which has the minimum parameters in DEIM series, is further selected as the benchmark model to improve training efficiency and control structural complexity.

**Figure 6:**
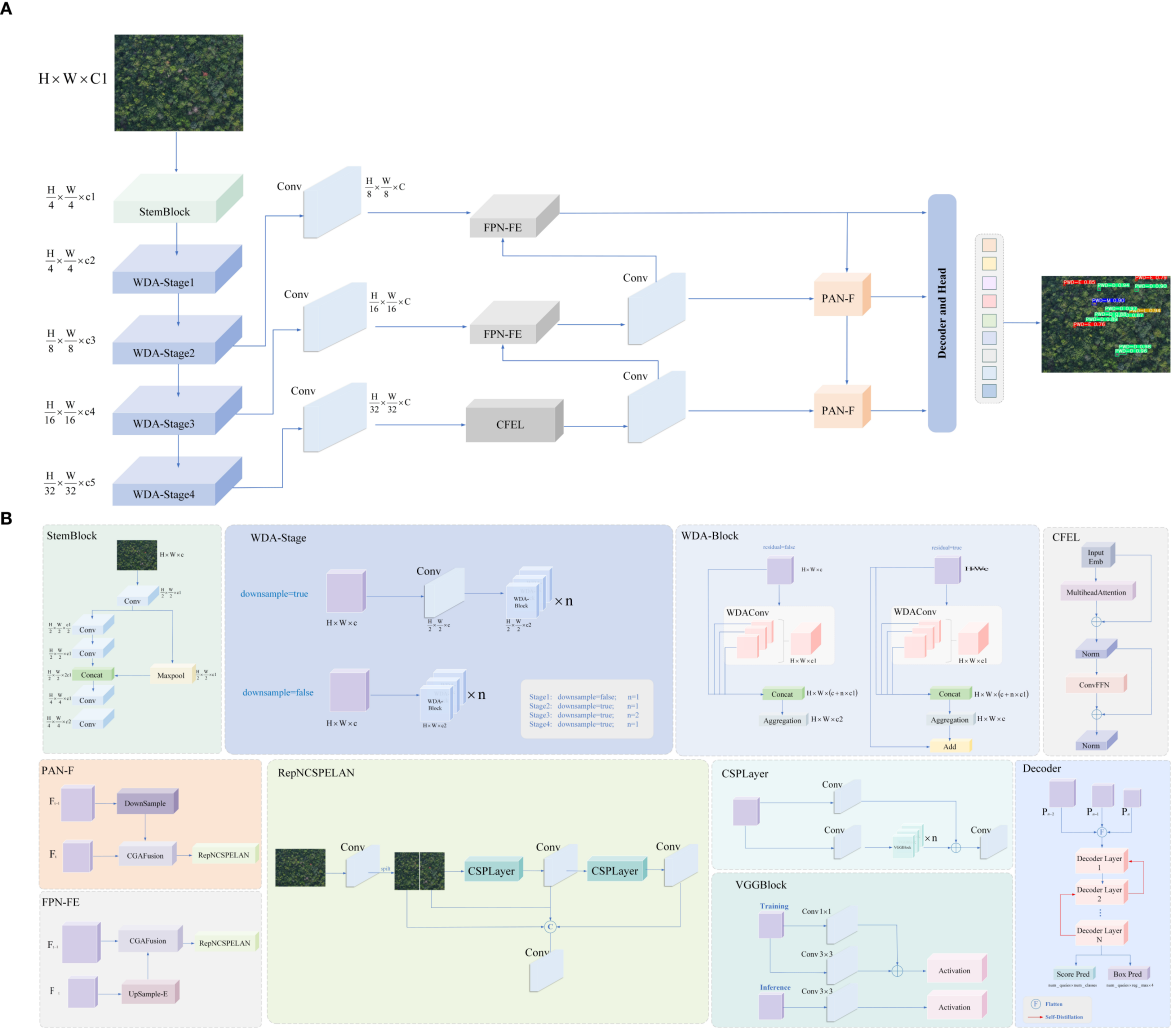
LE-PWDNet model structure framework. **(A)** Overall framework of LE-PWDNet. **(B)** Detailed structure of the proposed module.

Based on this training mechanism, this paper designs a lightweight detection model LE−PWDNet, whose structure is shown in **Figure 6**. Further, aiming at the problems of “weak texture”,”fuzzy structure” and “disjoint upper and lower semantics” in early disease spot detection, the network structure is systematically optimized from three key links: feature modeling, information fusion, and spatial reconstruction. In the feature extraction stage, a WDAConv is proposed, which combines wavelet transform, detail enhancement and parallel attention mechanism to enhance the perception ability of the model to lesion edges, local textures, and multi-scale structures. In addition, ConvFFN is introduced for high-frequency feature enhancement. By embedding local convolution branches in the spatial domain between two linear transformations, ConvFFN strengthens the model’s ability to capture fine-grained lesion edges and texture anomalies. In the feature fusion stage, CGAFusion module is constructed to realize cross-layer feature aggregation driven by semantic consistency through channel, spatial and pixel attention guidance; in the up-sampling and structure reconstruction stage, DySample-E module is designed to effectively enhance structural continuity modeling and suppress missed detection and boundary misjudgment of small targets by combining expansion convolution and edge residual guidance mechanism. These modules have their own emphasis on functional localization and cooperate to cover the complete detection process from perception enhancement to feature fusion to spatial reconstruction. On the premise of ensuring the overall parameter scale and computational overhead control, this structure aims to improve the recognition accuracy and robustness of the model to early PWD targets.

Overall, the proposed LE-PWDNet is not a parameter-reduced variant of existing lightweight detectors, but a task-specialized architecture explicitly designed for the weak-texture and small-scale characteristics of early PWD lesions. Its components operate in a complementary manner to address the intrinsic challenges of early-stage forest disease detection, including enhancing high-frequency perception, preserving subtle structural cues, enforcing cross-layer semantic alignment, and restoring fine-grained spatial continuity. As a result, the model achieves a level of detail sensitivity and robustness that conventional lightweight convolutional frameworks cannot attain under similar computational constraints.

### 2.4 Wavelet detail attention convolution

**Figure 7:**
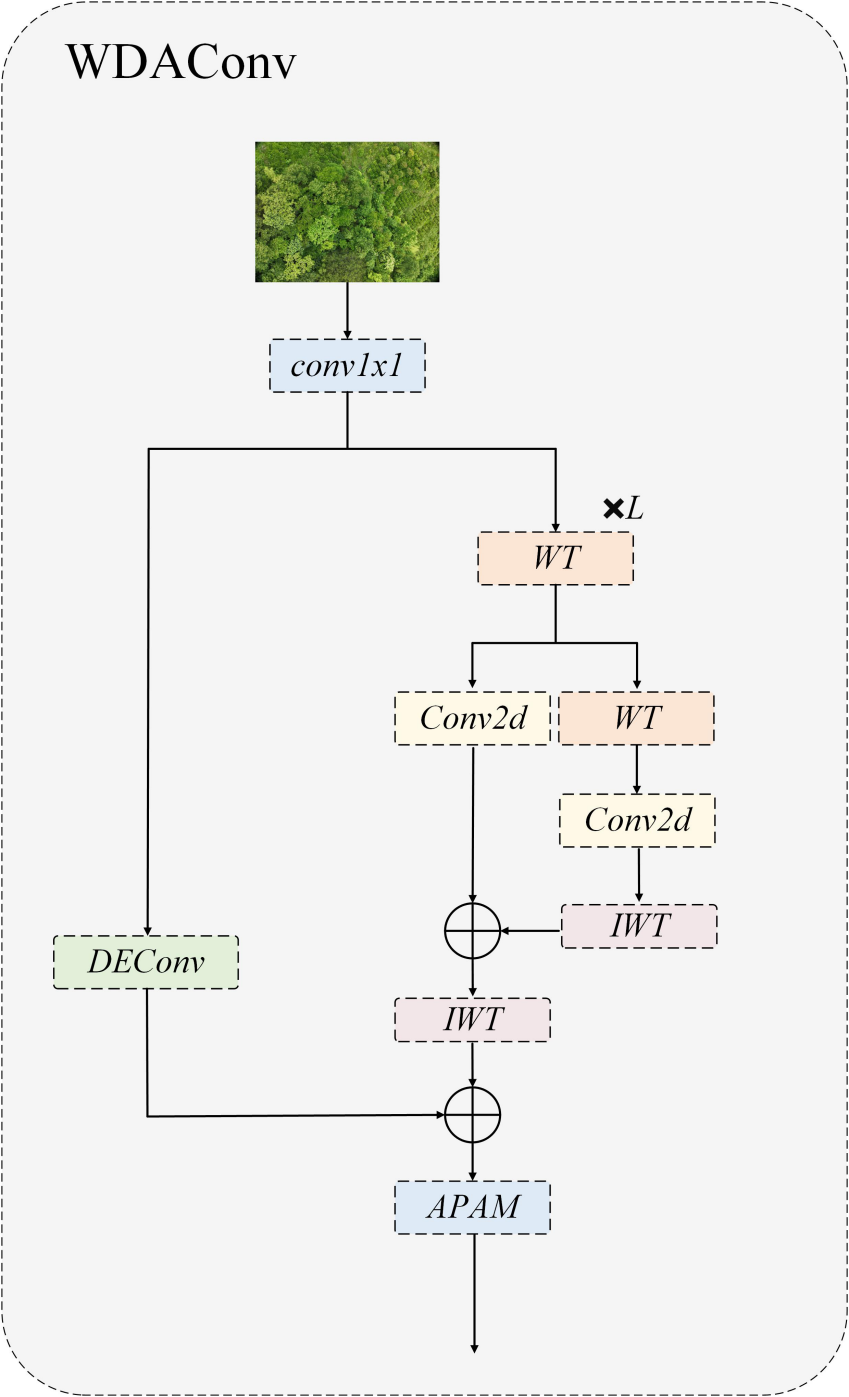
Structure of WDAConv.

The detection of early PWD presented a complex difficulty of small scale, weak contrast, dense and easy occlusion. The disease spots were mostly fine chlorosis and fine texture anomaly of needle level, which were essentially high frequency components and were often submerged by complex woodland background. If high frequency details are suppressed or lost in the early stage of feature extraction, the separability and detection stability of small targets will decrease significantly. In order to meet the above challenges, this research innovatively proposes Wavelet Detail Attention Convolution (WDAConv), which is a multipath convolution module around frequency decomposition, structure coding and saliency fusion, and its overall architecture are illustrated in **Figure 7**. The module uses 2D Haar wavelet to decompose and reconstruct multi-scale frequency (Finder et al., 2024), DEConv to encode multi-directional edge and texture, and APAM to implement joint reweighting in channel and spatial dimensions to complete cross-branch semantic consistent aggregation.

Specifically, the module first passes through a 1×1 convolution layer, which functions as a channel projection to align the dimensionality of subsequent branches without altering the spatial receptive field. Afterwards, a wavelet transform is employed to decompose the input image into multi-level frequency subbands (Wu et al., 2025), and convolution operations are applied independently to the separated low- and high-frequency components. Given an input image X, the WDAConv begins by conducting a two-dimensional Haar wavelet transform as the initial step. This transformation is realized through four filters, each employed to generate distinct directional and frequency subcomponents of the decomposition, as described in Equation 1.

(1) $$\begin{array}{c} \;f_{LL}=\frac{1}{2}\left[\begin{array}{cc} 1 & 1\\ 1 & 1 \end{array}\right],f_{LH}=\frac{1}{2}\left[\begin{array}{cc} 1 & -1\\ 1 & -1 \end{array}\right],f_{HL}=\frac{1}{2}\left[\begin{array}{cc} 1 & 1\\ -1 & -1 \end{array}\right],f_{HH}=\frac{1}{2}\left[\begin{array}{cc} 1 & -1\\ -1 & 1 \end{array}\right] \end{array}$$

Subsequently, the extracted frequency subbands are processed using depthwise convolutions with small kernels, as formulated in Equation 2.

(2) $$\begin{array}{c} \left[X_{LL},X_{LH},X_{HL},X_{HH}\right]=\text{Conv}\left(\left[\;f_{LL},f_{LH},f_{HL},f_{HH}\right],X\right) \end{array}$$

Equation 3 is employed to conduct the inverse wavelet transform, restoring the feature representation to its original spatial resolution. This operation integrates feature information from the various frequency channels to generate the final output. Subsequently, the convolved feature map of Y and DEConv is subjected to weighting.

(3) $$\begin{array}{c} Y=\text{IWT}\left(\text{Conv}\left(W,\text{WT}\left(X\right)\right)\right) \end{array}$$

Meanwhile, the parallel structural path uses the DEConv branch to encode high-frequency structures such as edges and textures in multiple directions. By leveraging reparameterization, it folds into a single standard convolution kernel during both training and inference stages, thereby controlling additional overhead while maintaining interface consistency. The two paths are aggregated via a residual connection and fed into the APAM. Finally, the fused feature maps undergo dynamic weighting across both channel and spatial dimensions via the Adaptive Parallel Attention Module (APAM), and the channel count is unified via vanilla convolution, resulting in a precise and highly discriminative comprehensive feature representation. Given the structural division of labor in the HGNetv2 backbone network, where shallow layers focus on local texture modelling and deep layers focus on global semantic abstraction, this study systematically introduces the WDAConv based on the original architecture to construct a new WDA Block structure. By embedding frequency-aware and detail-enhancing paths, the WDAConv enhances multi-scale feature representation and minimizes computational cost through independent Haar subband processing and DEConv reparameterization, particularly achieving effective retention of high-frequency textures and coordinated fusion of cross-scale semantics in shallow and intermediate layers. Furthermore, its high-frequency detail retention and robust modelling characteristics complement the dense matching training paradigm based on DEIM.

While maintaining a lightweight design, WDAConv is able to preserve weak-contrast details and needle-leaf directional textures that are easily lost by conventional spatial convolutions, standalone attention mechanisms, or pure wavelet convolutions. These existing methods often struggle to maintain both high-frequency fidelity and semantic consistency in weak-texture and low-contrast small-target scenarios. By alleviating early-stage feature degradation, WDAConv provides more reliable fine-grained cues for the stable recognition of early-stage PWD lesions. In essence, WDAConv offers a frequency-aware lightweight convolutional modeling approach tailored for weak-texture small-target detection, an ability that traditional spatial-domain convolutional structures inherently lack.

#### 2.4.1 DEConv

**Figure 8:**
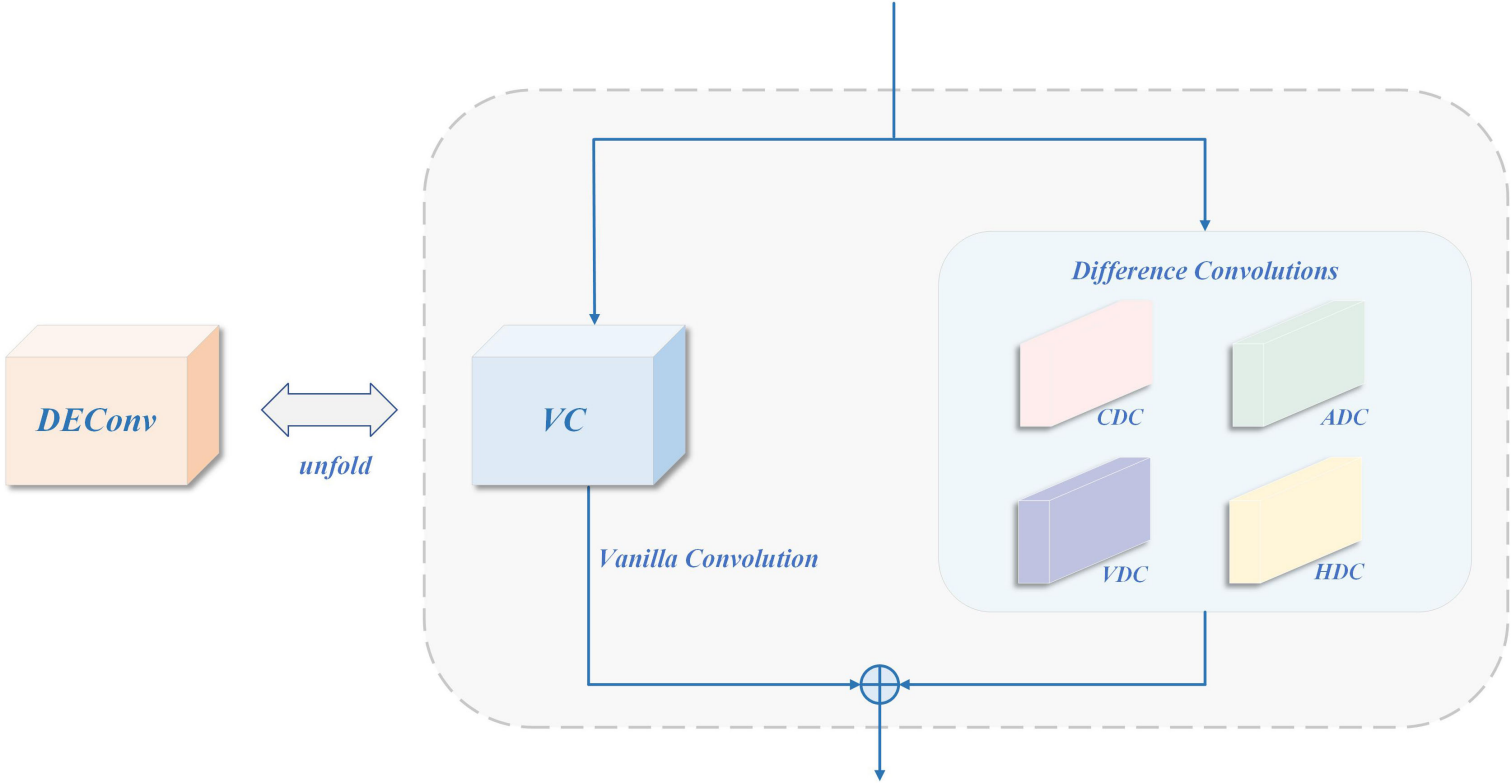
Structure of DEConv.

DEConv (Chen et al., 2024) is introduced as a compensation path for high-frequency details, aimed at strengthening the accurate characterization of fine-grained textures and edge structures. This module adopts multi-branch structure, which fuses standard convolution and differential convolution in multiple directions (such as vertical, horizontal, and diagonal) and can effectively capture weak texture anomalies and structural mutations in the lesion area. Its structure is shown in **Figure 8**. The fused features are subjected to channel aggregation through 1x1 convolution to output an enhanced feature map (Wang et al., 2024a; Chen and Wang, 2025; Zhao et al., 2025).

**Figure 9:**
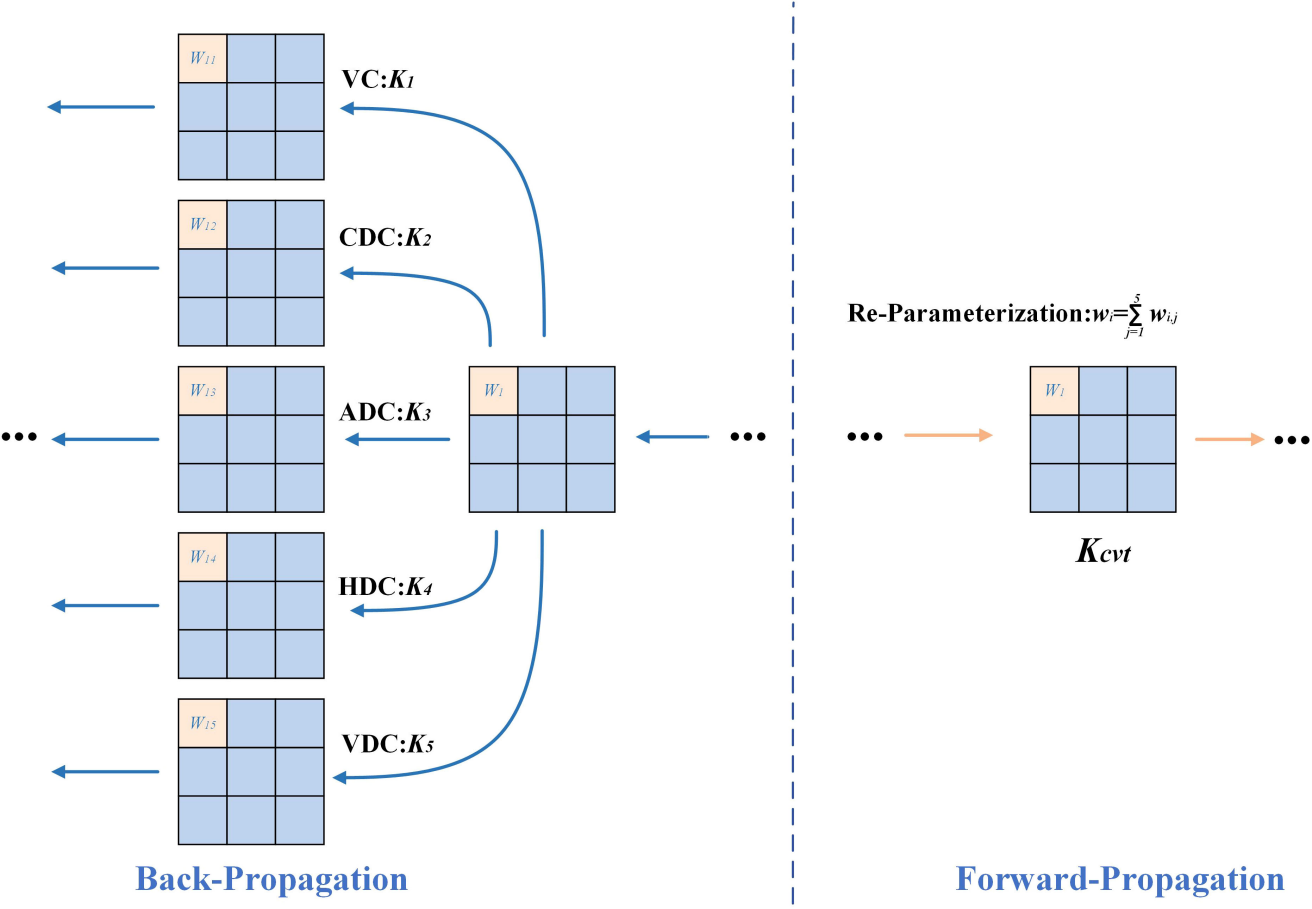
The structure of structural reparameterization in DEConv.

In order to reduce the computational overhead in the inference stage, DEConv adopts a reparameterization strategy to combine the convolution kernels of the five branches in the training stage into a single convolution kernel when propagating forward, as shown in **Figure 9**. The specific form is as follows, as defined in Equation 4:

(4) $$\begin{array}{c} \begin{array}{c} F_{out}=DEConv\left(F_{in}\right)=\sum_{i=1}^{5}F_{in}*K_{i}\\ =F_{in}*\left(\sum_{i=1}^{5}K_{i}\right)=F_{in}*K_{cvt}, \end{array} \end{array}$$

Where $K_{i=1:5}$ represents convolution kernels in different directions (including VC, HDC, VDC, CDC, ADC), * represents convolution operation, and $K_{i=cvt}$ is the equivalent convolution kernel after conversion.

This structure can improve the modeling ability of small target disease spots without increasing model parameters and calculation cost, especially suitable for disease detection tasks sensitive to high frequency details under resource-limited platforms.

#### 2.4.2 Adaptive parallel attention mechanism

Convolutional Block Attention Mechanism (CBAM) (Woo et al., 2018), as a lightweight and efficient attention mechanism module, plays a unique and key role in the field of deep learning. It has an excellent ability to perform Attention operations simultaneously in the spatial and channel dimensions to refine input features. Previously, ResNet and others focused only on channel dimensions and ignored key information about spatial dimensions. CBAM is different. In spatial dimension, Spatial Attention Module (SAM) can accurately locate key areas, so that the model can better focus on the target and improve the perception of image details. The Equation 5 is as follows:

(5) $$\begin{array}{c} F_{sa}=\sigma \left(f^{7\times 7}\left(\left[AvgPool\left(f_{in}\right);MaxPool\left(f_{in}\right)\right]\right)\right) \end{array}$$

where σ(·) denotes the Sigmoid activation function, while $f^{7\times 7}\left(\cdot \right)$ indicates a two-dimensional convolution with a 7×7 kernel, followed by batch normalization and a ReLU activation. $F_{sa}$ corresponds to the feature map generated by SAM.

In the channel dimension, the Channel Attention Module (CAM) can adaptively adjust weights to highlight critical channels and suppress redundancy. The Equation 6 can be expressed as

(6) $$\begin{array}{c} F_{ca}=\sigma \left(MLP\left(AvgPool\left(f_{in}\right)\right)+MLP\left(MaxPool\left(f_{in}\right)\right)\right) \end{array}$$

where σ(·) is used to denote the Sigmoid activation, $f_{in}$ indicates the input feature map, and $F_{ca}$ designates the feature representation produced by CAM.

Experiments show that CBAM-based model performs better than channel-only ResNet in image classification, detection, and segmentation, and can improve feature utilization efficiency on MobileNet, showing universality and strong performance.

**Figure 10:**
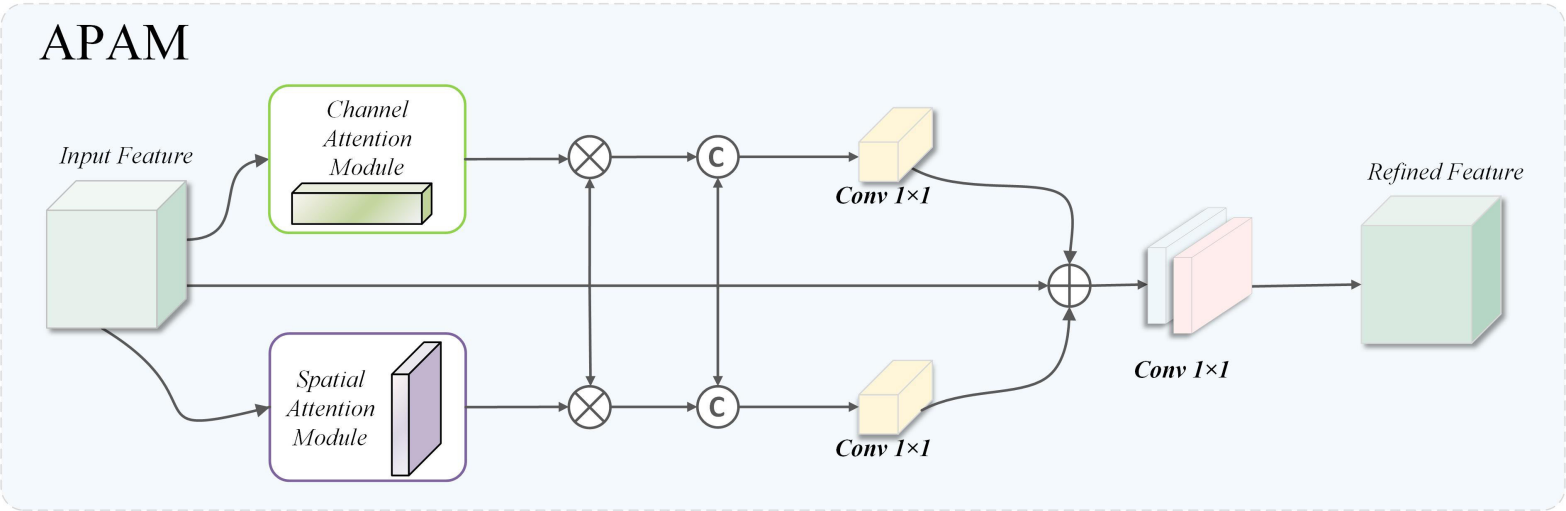
Structure of APAM.

Designed to strengthen the model’s recognition of early pine wood nematode traits in complex backgrounds, inspired by convolutional block attention mechanism (CBAM), this paper introduces a fusion attention module based on three-branch structure, namely adaptive parallel attention Mechanism (APAM), whose structure is shown in the **Figure 10**. Based on the original feature path, this module introduces channel guidance branch and space guidance branch and realizes complementary enhancement of multidimensional features through hopping connection and fusion mechanism. Specifically, the original feature map is refined by CAM and SAM to extract channel- and spatial-level information, which is subsequently multiplied with the original features to preserve dimensional consistency. The equations corresponding to this process are given in Equations 7–9:

(7) $$\begin{array}{c} f_{c}=\left[f_{in};CAM\left(f_{in}\right)⊗f_{in}\right] \end{array}$$

(8) $$\begin{array}{c} f_{s}=\left[\;f_{in};SAM\left(f_{in}\right)⊗f_{in}\right] \end{array}$$

(9) $$\begin{array}{c} f_{out}=f^{1\times 1}\left(f^{1\times 1}\left(f_{in}+f^{1\times 1}\left(f_{c}\right)+f^{1\times 1}\left(f_{s}\right)\right)\right) \end{array}$$

where $f^{1\times 1}\left(\cdot \right)$ indicates a two-dimensional convolution with a 1×1 kernel combined with batch normalization and ReLU activation, CAM denotes the channel attention mechanism, and SAM denotes the spatial attention mechanism.

The proposed APAM can model collaboratively in three dimensions: channel, space, and original features, and strengthen the ability of capturing significant features of disease regions while maintaining original semantic information. It can significantly improve the perception accuracy and regional positioning ability of weak disease spots in early PWD detection, especially suitable for complex scenes with small lesion area, fuzzy boundary or covered by background interference.

This parallel formulation preserves the semantic integrity of the input while enabling complementary enhancement from multiple attention dimensions. More importantly, APAM is tailored for weak-texture and low-contrast PWD lesions, where direct attenuation of fine-grained cues often occurs. By jointly modeling channel cues, spatial localization signals, and unaltered original textures, APAM effectively strengthens the representation of subtle diseased regions and mitigates feature dilution under complex canopy backgrounds. In addition, APAM aligns naturally with the frequency-enhanced outputs of WDAConv, providing cross-domain consistency between frequency-domain detail retention and spatial-domain semantic refinement, which is difficult to achieve using existing attention modules such as CBAM, SE, ECA, or CoordAtt.

### 2.5 ConvFFN

**Figure 11:**
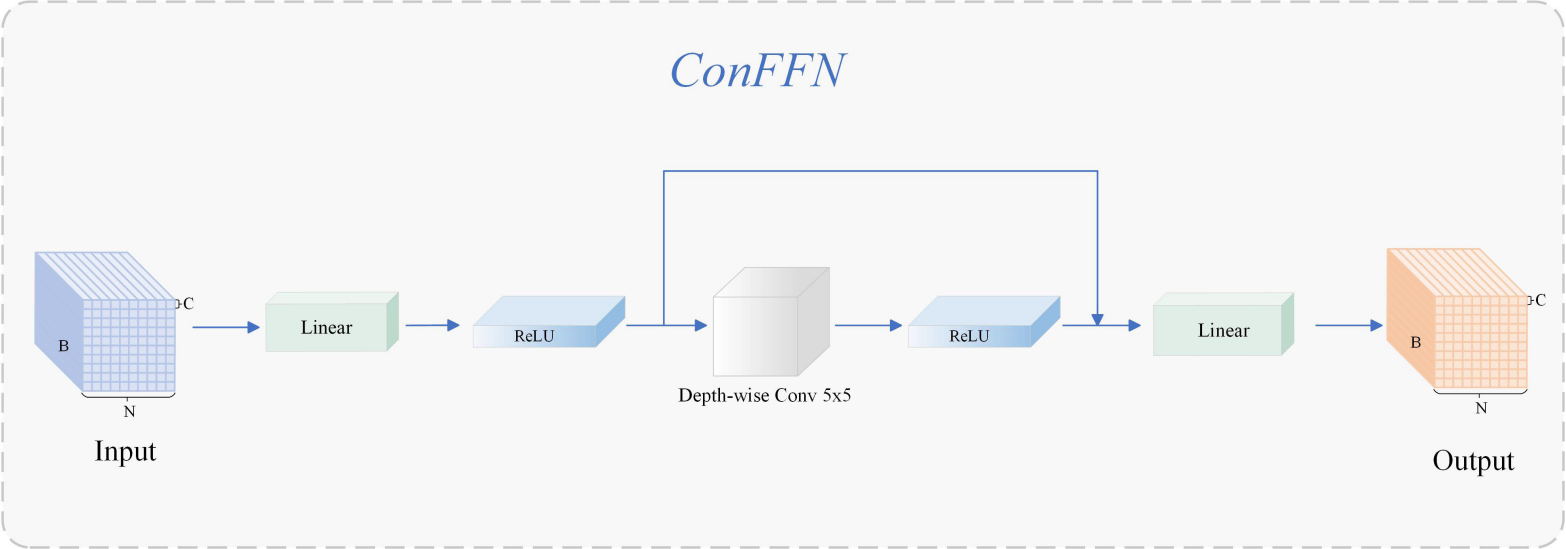
Structure of ConFFN.

Although Vision Transformer exhibits strong modeling capabilities for visual tasks, its inherent low-pass filtering properties make it less responsive to local high-frequency structures, especially for detecting high-frequency pathological features such as early PWN lesions that present weak chlorosis and fine texture. For this structural shortcoming, this paper introduces ConvFFN (Zhou et al., 2023a, b) to improve the modeling ability of encoder for fine-grained texture. ConvFFN structure is shown in **Figure 11**. This module was first proposed by Zhou et al. (2023a) in SRFormer and subsequently developed into a lightweight high-frequency modeling scheme in CATANet (Liu et al., 2025a). ConvFFN achieves spatial dimension information enhancement while maintaining FFN interface consistency by embedding 5×5 convolution branches with depth separation between two linear transforms, effectively alleviating self-attention suppression of high-frequency components (Chenghai et al., 2025).

Different from the previous “channel-dominated” FFN structure, ConvFFN provides a high-frequency compensation paradigm: it introduces local convolution paths in the spatial domain to accurately capture abnormal patterns such as lesion edges and texture disturbances, especially suitable for complex environments where needle-scale details are seriously disturbed by woodland background in early PWN detection. ConvFFN provides an ideal scheme for forest disease early detection on edge equipment, which considers both accuracy and efficiency. In this study, the high-frequency enhancement mechanism is introduced into the Transformer structure for PWN detection for the first time, which not only improves the expression ability of the model for high-frequency susceptible regions, but also provides more discriminative underlying support for the subsequent feature fusion module.

### 2.6 CGAFusion

Early-stage PWD lesions typically exhibit subtle chlorosis and fine texture disruptions, which are easily obscured by complex forest backgrounds. Shallow features, though rich in detail, often degrade through downsampling, while base fusion with deep features may cause semantic misalignment and result in missed detections.

**Figure 12:**
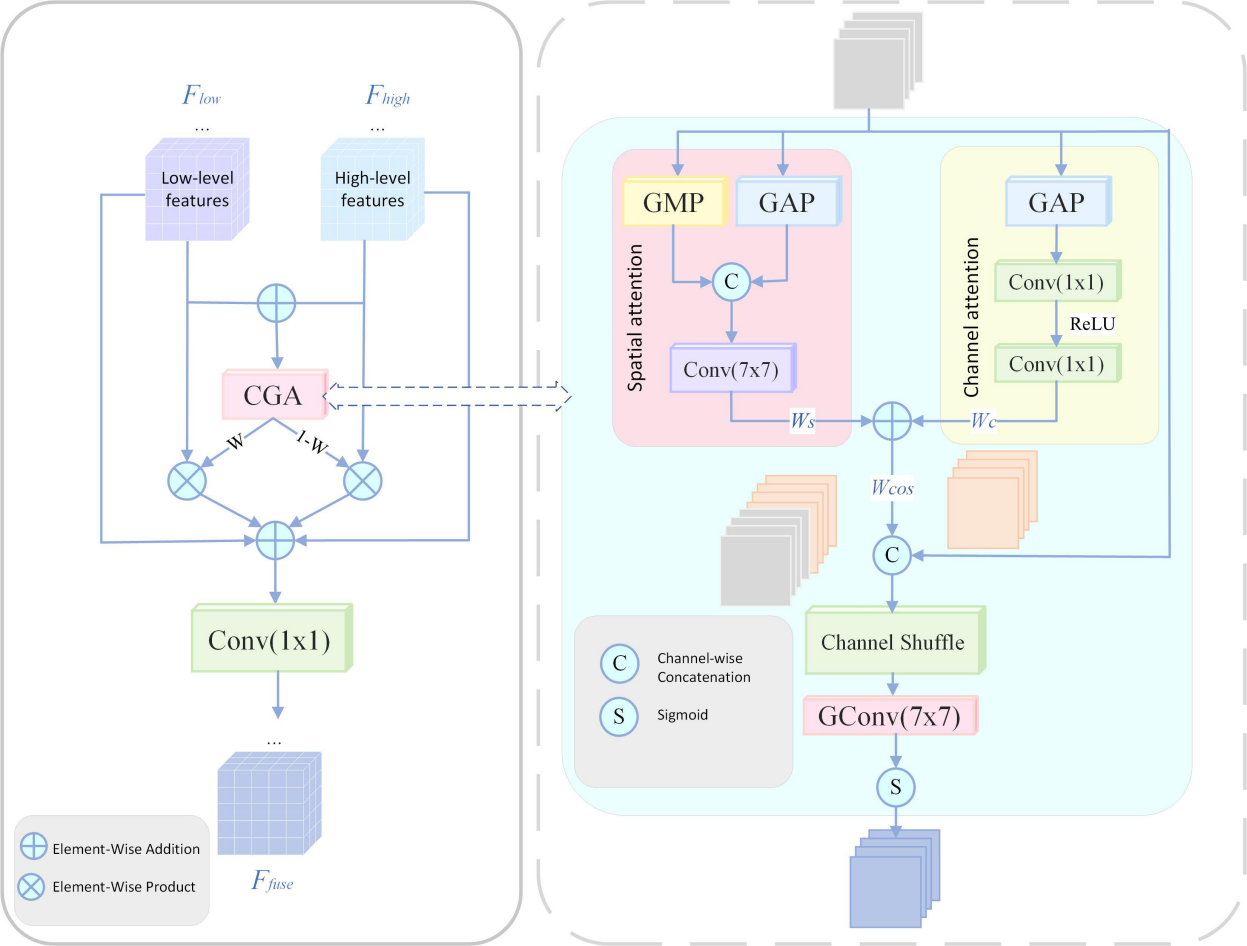
Structure of CGAFusion.

To mitigate this, the proposed Content-Guided Attention Fusion (CGAFusion) (Chen et al., 2024) module leverages a coarse-to-fine, content-driven attention mechanism across channel, spatial, and pixel levels. Its structure is shown in **Figure 12**. By generating Saliency Importance Maps (SIMs) and applying residual-enhanced weighted fusion (Li et al., 2025; Ni et al., 2025), CGAFusion reinforces discriminative regions while maintaining stable feature propagation, thus improving the detection of weak, small-scale targets.

The corresponding fusion equation is given in Equation 10:

(10) $$\begin{array}{c} F_{fuse}=C_{1\times 1}\left(F_{low}\cdot W+F_{high}\cdot \left(1-W\right)+F_{low}+F_{high}\right) \end{array}$$

where $F_{low}$ and $F_{high}\;$ represent features from different levels; W is the spatial attention weight map generated by the CGAFusion; the weighted sum also introduces a residual connection $F_{low}+F_{high}$ to improve training stability and information transmission capabilities.

Its coarse-grained attention is obtained by adding channel attention $W_{c}$ and spatial attention $W_{s}$, as defined in Equation 11:

(11) $$\begin{array}{c} W_{\text{coa}}=W_{c}+W_{s} \end{array}$$

Finally, in order to obtain the refined global feature map $SIM_{s}W$, the CGAFusion adjusts each channel of $W_{\text{coa}}\in {\mathbb{R}}^{C\times H\times W}$ under the guidance of the input features to generate the final $SIM_{s}W$. The operation process is described in Equation 12:

(12) $$\begin{array}{c} W=\sigma \left(\vartheta C_{7\times 7}\left(CS\left(\left[X,W_{\text{coa}}\right]\right)\right)\right) \end{array}$$

here σ denotes S-type operation, CS· denotes channel mixing operation. CGAFusion assigns specific spatial importance maps (SIMs) to each channel, guiding attention toward the most informative regions and enhancing feature discrimination (Wang et al., 2024b; Chen and Hao, 2025; Liu et al., 2025b). Building on this, the CGAFusion module integrates semantic guidance with structural alignment to highlight lesion areas, suppress background noise, and achieve robust multi-scale fusion, thereby improving detection accuracy for early, subtle symptoms.

### 2.7 DySample-E

In the original HybridEncoder architecture, the upsampling operations in the FPN path typically employ non-parametric methods such as nearest-neighbor interpolation. While these interpolation methods have low computational overhead, their receptive field is fixed to a single pixel, which poses significant limitations in tasks requiring extremely fine-grained perception, such as early detection of PWD. When the target region exhibits mild de-greening and weak texture perturbations and occupies only a very small number of pixels, non-learning-based interpolation amplifies these already weak details into structural noise, disrupting edge continuity and causing gradient instability, ultimately leading to false negatives and false positives in small target regions.

**Figure 13:**
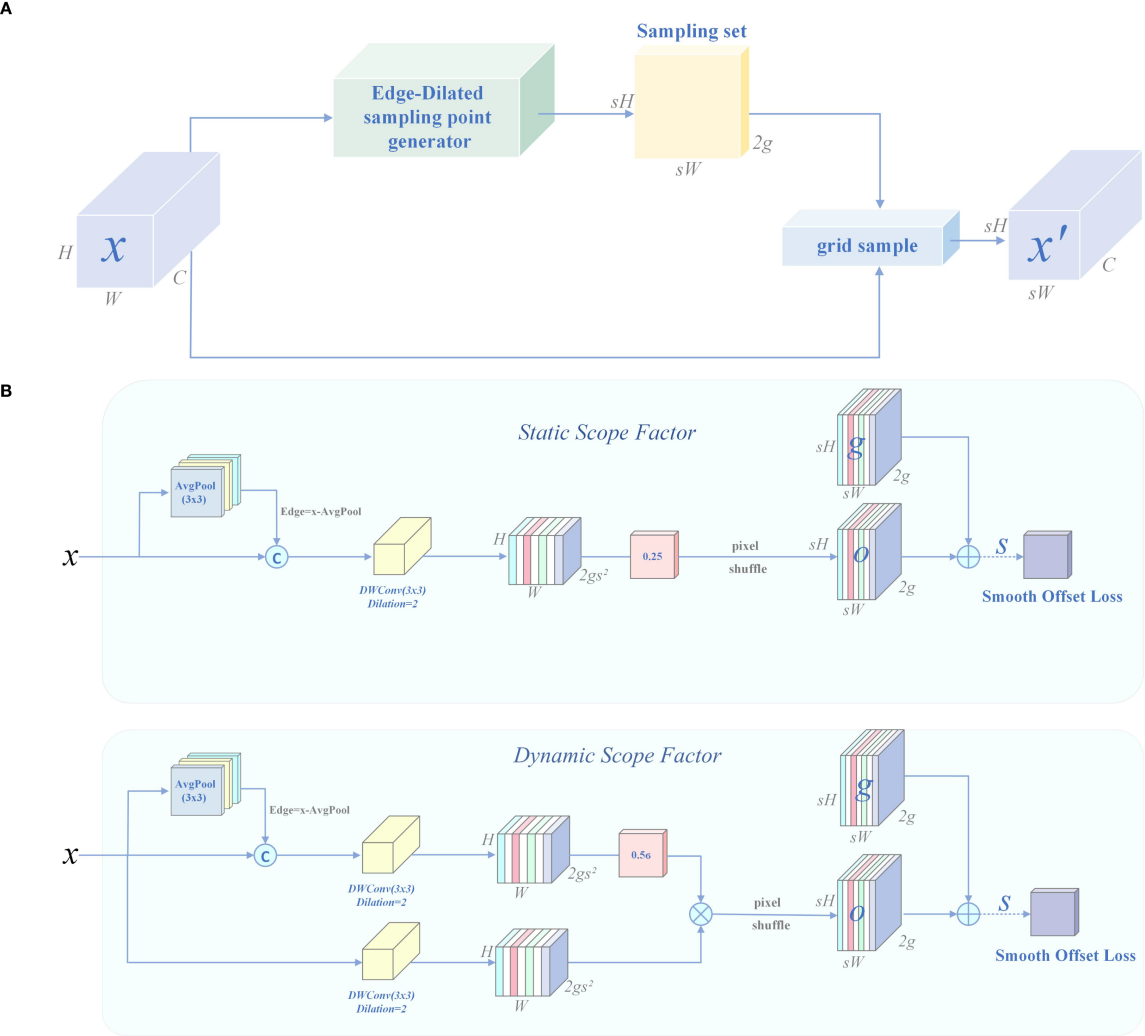
Structure of DySample-E. **(A)** Sampling based dynamic upsampling. **(B)** Edge-Dilated sampling-point generator (DySample-E).

To overcome the above bottlenecks, this paper proposes a structurally optimized lightweight dynamic upsampling module, DySample-E (Edge-Dilated sampling-point generator), whose structure is shown in **Figure 13**. This module is embedded in the feature backpropagation path to enhance detail recovery capabilities. DySample-E follows the DySample (Liu et al., 2023c) framework in structure, as shown in Equation 13, and performs learnable spatial sampling based on the offset field.

(13) $$X_{\left(c,\tilde{h},\tilde{w}\right)}^{'}=\sum_{k=1}^{K}w_{\left(c,k,\tilde{h},\tilde{w}\right)}\cdot X_{\left(c,\left[\frac{\bar{h}}{s}\right]+\Delta y_{k},\left[\frac{\bar{w}}{s}\right]+\Delta x_{k}\right)}$$

Where  s  is the amplification factor, ${\left\{\text{Δ}x_{k},\text{Δ}y_{k}\right\}}_{k=1}^{K}$ is the sampling offset set, dynamically generated by the sampling-point generator based on the input feature X, and the weight w(·) is implicitly derived from the bilinear kernel of the grid sample function, eliminating the need for explicit convolution calculations.

The innovation of DySample-E lies in the introduction of an edge residual guidance mechanism, which obtains high-frequency edge information through the difference between the input feature map X and its 3×3 average pooling map, as defined in Equation 14:

(14) $$\begin{array}{c} \text{E}=\text{X}-\text{AvgPoo}{\text{l}}_{3\times 3}\left(\text{X}\right) \end{array}$$

The edge residual feature E is concatenated with the original feature X as the input to the offset prediction network, which explicitly focuses on microtexture changes such as needle edges and disease spot boundaries.

Subsequently, DySample-E uses a dual-branch structure and dilated depth-separable convolution (DWConv) to extract offset information, thereby expanding the receptive field to 5×5 without increasing the computational load, as defined in Equations 15, 16:

(15) $$\begin{array}{c} {\text{O}}_{s}=\text{DWCon}{\text{v}}_{3\times 3}^{d=2}\left(\left[\text{X}∥\text{E}\right]\right) \end{array}$$

(16) $$\begin{array}{c} {\text{O}}_{d}=\text{DWCon}{\text{v}}_{3\times 3}^{d=2}\left(\left[\text{X}∥\text{E}\right]\right) \end{array}$$

Where ${\text{O}}_{s}$ is the static branch output, and ${\text{O}}_{d}$ is the dynamic gate output, which predict the static and adaptive offsets, respectively.

To generate the final two-dimensional displacement field Δ=(Δx,Δy), DySample-E introduces a static/dynamic sampling factor fusion mechanism. The static displacement is generated by ${\text{O}}_{s}$, as defined in Equation 17:

(17) $$\begin{array}{c} {\text{Δ}}_{s}={\text{PixelShuffle}}_{s}\left(0.25\cdot {\text{O}}_{s}\right) \end{array}$$

The dynamic offset is modulated by the global channel attention control weight λ=σ(GAP(X)) and calculated based on ${\text{O}}_{d}$, as defined in Equation 18:

(18) $$\begin{array}{c} {\text{Δ}}_{d}={\text{PixelShuffle}}_{s}\left(\lambda ⊗{\text{O}}_{d}\right),\text{Δ}={\text{Δ}}_{s}+{\text{Δ}}_{d} \end{array}$$

Finally, the upsampling operation is performed using grid sampling, as defined in Equation 19:

(19) $$\begin{array}{c} {\text{X}}^{\prime }=\text{grid sample}\left(\text{X},\text{Δ}\right) \end{array}$$

To alleviate checkerboard artifacts caused by dynamic displacement and improve training stability, a total variation regularization term was introduced, as defined in Equation 20, to smooth the displacement field:

(20) $$\begin{array}{c} {ℒ}_{smooth}=∥\nabla \text{Δ}{∥}_{1} \end{array}$$

In summary, DySample-E successfully upgrades traditional static interpolation upscaling to a structure-sensitive, semantically adaptive dynamic reconstruction process by combining edge-guided modelling, dual-branch offset prediction, and explicit spatial sampling. This module achieves high-quality upscaling of small-scale weak targets without introducing additional convolution kernels or redundant parameters, providing more discriminative and robust high-resolution feature support for the precise detection of early-stage PWD in complex forest areas.

## 3 Results

### 3.1 Experiment setting

#### 3.1.1 Experimental setup and parameters

All experiments in this study were carried out within a unified hardware and software setup to guarantee the stability and reproducibility of the results, with detailed configurations provided in **Table 1**. Before model training, a complete set of hyperparameter schemes was pre-set, including learning rate, batch size, optimizer, and training rounds, to promote sufficient convergence and performance improvement of the model. The detailed training parameter configuration is shown in **Table 2**.

**Table 1:** Experimental environment parameters.

Experimental environment	Detailed parameters
CPU	Intel Core i7-14700HX
GPU	NVIDIA RTX 4070 Laptop (8 GB)
RAM	16GB
Operating System	Windows11
Programming Language	Python 3.10
Deep Learning Framework	Pytorch2.3.1
CUDA	11.8
cuDNN	8.9.0

**Table 2:** Unified training hyperparameters for all experiments.

Training settings	Detailed parameters
Input image size	640×640
Batch Size	8
Epochs	300
Initial learning rate	0.0008
Optimizer	AdamW
Weight Decay	0.0004

#### 3.1.2 Evaluation metrics

Given the comprehensive requirements of PWD detection in terms of accuracy, efficiency, and deployability, this study conducts a multi-dimensional evaluation of the proposed model. The evaluation metrics include Average Precision (AP), the number of parameters (Params), giga floating-point operations (GFLOPs), and frames per second (FPS).

Specifically, Params represent the total number of trainable weights and biases in the model, serving as an important indicator of memory and storage requirements. GFLOPs measure the computational complexity of the model; higher values indicate greater computational cost during inference. FPS reflects the model’s real-time processing capability, a critical consideration for timely disease monitoring in UAV applications.

Among all these metrics, Average Precision (AP) is considered the most important indicator of detection accuracy. It is calculated as the area under the precision-recall curve, offering a comprehensive evaluation of the model’s performance across varying confidence thresholds. The specific formulae used to compute these metrics are presented below, as defined in Equations 21–23.

(21) $$\begin{array}{c} Precision=\frac{TP}{TP+FP} \end{array}$$

(22) $$\begin{array}{c} Recall=\frac{TP}{TP+FN} \end{array}$$

(23) $$\begin{array}{c} AP=\int_{0}^{1}P\left(r\right)dr \end{array}$$

True Positive (TP) is defined as the number of PWD targets that are correctly identified, whereas False Positive (FP) corresponds to the number of non-PWD instances that are mistakenly classified as PWD. False Negative (FN) indicates the count of actual PWD targets that remain undetected by the model. In addition, ${\text{AP}}_{50}$ and ${\text{AP}}_{50:95}$ represent the Average Precision values calculated at an IoU threshold of 0.50 and over the interval from 0.50 to 0.95, respectively, thereby offering a more thorough assessment of detection accuracy under different localization constraints.

### 3.2 Comparison experiments of different models

To provide a comprehensive assessment of Model LE-PWDNet, this paper selected the current mainstream object detection algorithms for comparison experiments. These include SSD (Liu et al., 2016), Faster R-CNN (Girshick, 2015), YOLOv8n (Yaseen, 2024), YOLOv12n (Tian et al., 2025), MobleNetv3 (Koonce, 2021), ShuffleNetv2 (Ma et al., 2018), RTDETRV2 and DEIM. The results of the comparative experiment are shown in **Table 3**. Multiple independent runs were conducted for all models to reduce the impact of randomness. To highlight the stability of the proposed model under repeated training, only the mean ± standard deviation of LE-PWDNet’s AP_50_ is reported in the table, while the other comparison models are presented using their averaged AP_50_ results.

**Table 3:** Comparative experimental results of different models.

Models	$AP_{50}\left(\%\right)$					Params(M)	GFLOPs	FPS
	PWD-E	PWD-M	PWD-L	PWD-D	All			
SSD	63.5	70.9	76.3	74.4	71.3	5.65	1.58	130
Faster-RCNN	77.8	84.7	89.6	85.5	84.4	113	24.69	48
YOLOv8n	74.6	83.1	90.2	83.8	82.9	3.05	8.14	113
YOLOv12n	75.1	83.3	89.6	81.4	82.4	2.51	5.82	129
MobleNetv3	74.6	82.9	90.1	84.2	82.7	2.50	7.21	118
ShuffleNetv2	73.8	83.1	89.5	83.7	82.5	4.14	5.60	125
RTDETRV2-R18	79.1	82.8	91.6	86.4	85.0	24.49	59.82	69
DEIM-D-Fine-N	76.9	86.2	87.5	82.6	83.3	5.32	7.01	120
**LE-PWDNet**	**83.8 (± 0.15)**	**92.4 (± 0.1)**	**94.0 ± 0.1)**	**90.5 (± 0.2)**	**90.2 (± 0.14)**	**5.64**	**7.08**	**125**

Without a significant increase in model complexity, the improved model proposed in this study achieves stable and comprehensive performance gains in PWD detection, with particularly notable improvements in the early stages of infection. Compared with the lightweight baseline model DEIM-D-Fine-N, the proposed model adds only approximately 0.32 M parameters and 0.07 GFLOPs of computational cost. The detection accuracy in the early stage (PWD-E) increases markedly from 76.9% to 83.8%, indicating that the designed structural enhancements effectively strengthen the perception and representation of subtle features in early lesions, rather than relying on parameter stacking to achieve inflated performance. Meanwhile, the overall detection accuracy AP_50_ improves from the baseline 83.3% to 90.2%, underscoring the model’s detection advantages across the full infection cycle and better aligning with the urgent demand for early, high-precision identification in forestry pest control.

When compared with current mainstream lightweight detectors under the constraint of fewer than 6 million parameters, YOLOv8n, YOLOv12n, MobileNetV3, and ShuffleNetV2 achieve overall AP_50_ values of 82.9%, 82.4%, 82.7%, and 82.5%, respectively. Among them, YOLOv8n delivers relatively high detection accuracy but requires a computational cost of 8.14 GFLOPs; YOLOv12n reduces the computational load to 5.8 GFLOPs but achieves only 75.1% AP_50_ in the early stage. MobileNetV3 and ShuffleNetV2 have computational costs of 7.21 GFLOPs and 5.6 GFLOPs, respectively, which are relatively low, but their early-stage accuracies are only 74.6% and 73.8%, limiting their utility in early prevention and control. In contrast, the proposed model achieves an overall AP_50_ of 90.2% with only about 7.08 GFLOPs, and 83.8% AP_50_ in the early stage, both higher than all the above lightweight detectors and the DEIM baseline, fully demonstrating its favorable balance between accuracy and efficiency.

When compared with the high-capacity two-stage model Faster R-CNN and the Transformer-based RT-DETR V2-R18, although their overall accuracies reach 84.4% and 85.0%, respectively, their computational requirements are as high as 24.69 and 59.82 GFLOPs, respectively, significantly exceeding those of the proposed model. Notably, while RT-DETR achieved the highest single score of 91.6% in the late stage (PWD-L), its detection accuracy in the early infection stage (PWD-E) was 79.1%, still lower than the proposed model’s 83.8%. Additionally, in the severely degraded dead tree stage (PWD-D), the model proposed in this paper maintains a high detection performance of 90.5%, indicating that lightweight designs tailored for specific application domains can outperform general-purpose large-scale architectures in specific tasks. This highlights its robust identification and generalization capabilities for infected plants at all stages, particularly for early-stage lesions.

In terms of inference speed, the proposed model achieves 125 FPS, which is comparable to mainstream lightweight detectors such as YOLOv12n at 129 FPS and ShuffleNetV2 at 125 FPS, and is significantly faster than large-scale frameworks such as Faster R-CNN with 48 FPS and RT-DETR V2-R18 with 69 FPS. Although models such as YOLOv12, ShuffleNetV2, and SSD show slightly higher FPS, their detection accuracy in the early stage is notably insufficient, making them inadequate for identifying subtle lesions of PWD. In contrast, the proposed model not only maintains high inference efficiency but also delivers superior detection accuracy, ensuring a favorable compromise between computation efficiency and early-stage detection performance.

In summary, the lightweight detection framework LE-PWDNet proposed in this paper achieves a significant improvement in detection performance for the early infection stage of PWD without significantly increasing the number of parameters or computational overhead. The model strikes an effective balance between accuracy and lightweight design, meeting the real-time inference requirements of edge computing devices.

### 3.3 Ablation experiments of the proposed WDAConv

#### 3.3.1 Comparative experiments of different convolutional modules

To validate the effectiveness of the proposed WDAConv, this study conduct a series of comparative experiments by replacing the convolutional layers within each WDA-Block of the HGNetv2 backbone using various alternative modules. Specifically, this study compare the following variants: WDAConv、StarConv (Liu et al., 2022)、WTConv、PConv (Park et al., 2022) 、ARConv (Wang et al., 2025), with the results summarized in **Table 4**. To reduce stochastic variability, all convolutional modules were trained multiple times with different random seeds. To highlight the stability of the proposed module, **Table 4** reports only the mean ± standard deviation of WDAConv’s AP_50_ and AP_50_:_95_, while other variants are shown using their averaged results.

**Table 4:** Comparative experimental results of different convolutions.

Module	$AP_{50}\left(\%\right)$	$AP_{50:95}\left(\%\right)$	Params(M)	GFLOPs
Basic	82.2	57.3	5.32	7.01
StarConv	83.1	58.6	5.36	7.76
PConv	81.6	53.5	5.30	6.47
ARConv	82.7	58.5	6.79	10.98
WTConv	82.9	59.4	5.29	6.37
WDAConv	**83.7 (± 0.1)**	**61.1 (± 0.1)**	**5.43**	**7.05**

Comparative results show that WDAConv is the most performance- and efficiency-optimized convolutional alternative among all candidates. With only a modest increase of 0.11 M parameters and 0.04 GFLOPs, the model’s AP_50_ and AP_50_:_95_ are improved to 83.7% and 61.1%, placing WDAConv in a relatively advantageous position in the accuracy–efficiency trade-off. Although WTConv, which adopts the same wavelet-based frequency-domain design, achieves the lowest computational cost of 6.37 GFLOPs, its accuracy still lags behind WDAConv, with an AP_50_:_95_ shortfall of 1.7 percentage points, making it suitable only for extremely resource-constrained hardware scenarios. StarConv and ARConv deliver accuracy gains of less than 1 percentage point but incur an additional computational cost of 0.75 to 3.97 GFLOPs, leading to suboptimal performance for lightweight deployment. Meanwhile, PConv suffers from accuracy degradation due to impaired high-frequency feature preservation. In summary, WDAConv achieves a favorable balance between early-stage PWD detection and model efficiency by integrating wavelet-based multi-scale analysis, structural detail enhancement, and parallel attention mechanisms, making it the most effective replacement for standard convolution within the HGNetv2 backbone.

To further validate the quantitative superiority of WDAConv, we perform qualitative visualizations using EigenCAM (Muhammad and Yeasin, 2020). These visualizations focus on a representative early-stage PWD image, where subtle and low-contrast lesions are particularly challenging to detect. By comparing the activation maps produced by different convolutional modules under the same input condition, we aim to assess each module’s ability to capture fine-grained pathological cues.

**Figure 14:**
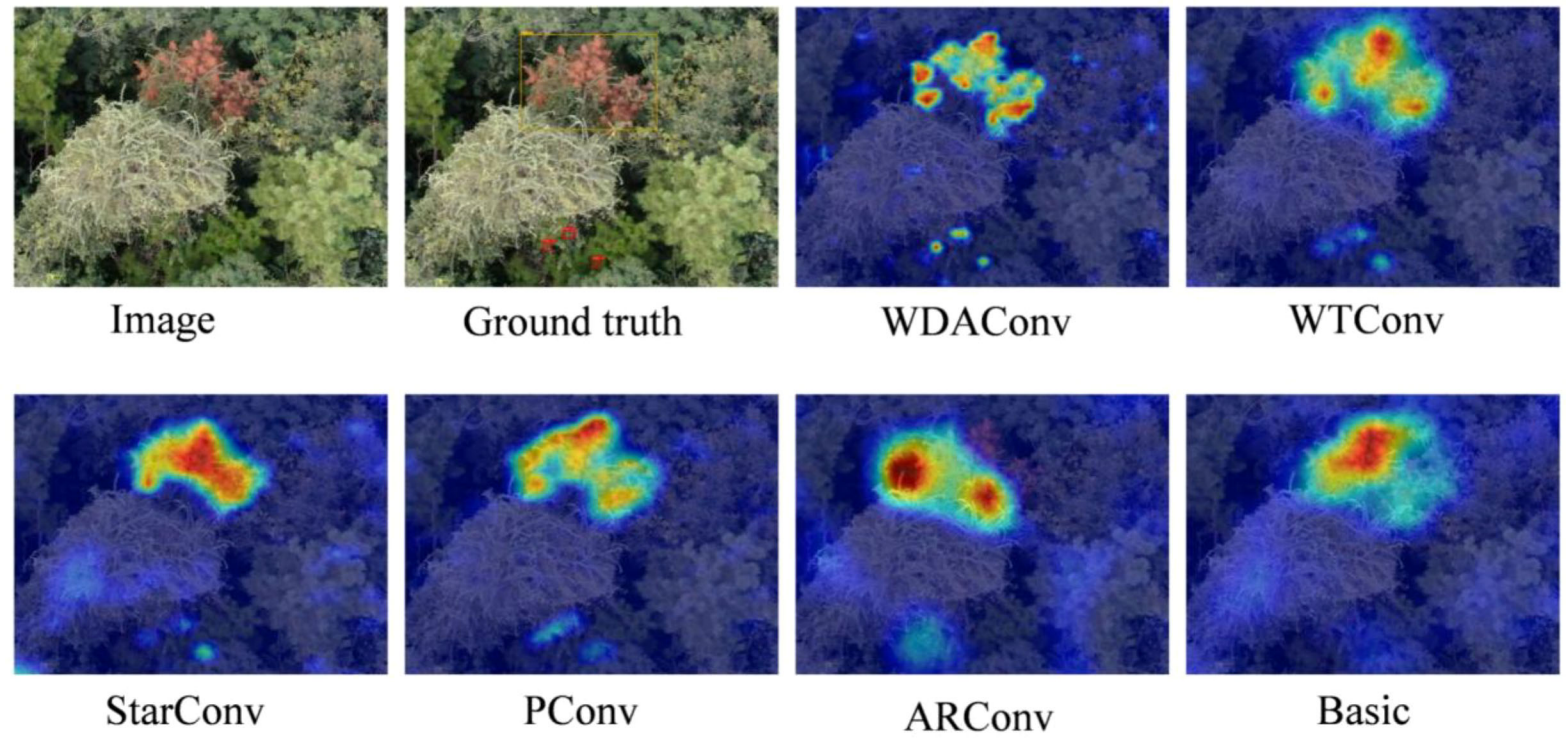
Comparative EigenCAM visualizations of different convolution modules.

As shown in **Figure 14**, based on the EigenCAM visualizations of the same early-stage sample image, the WDAConv demonstrates superior performance compared to Basic, StarConv, PConv, ARConv, and WTCConv. Specifically, WDAConv produces continuous and fine-grained high responses along the needle direction at two low-contrast early lesion areas, and maintains better boundary alignment and texture representation across other targets. In contrast, the other convolution strategies generally exhibit insufficient or fragmented responses in early-stage regions and coarse, blocky activations in late-stage lesions, making it difficult to capture subtle disease features accurately. This advantage aligns closely with the architectural design of WDAConv. By leveraging Haar wavelet decomposition to preserve multi-scale high-frequency information, DEConv for multi-directional structural encoding, and APAM for joint reweighting across channel and spatial dimensions, WDAConv effectively enhances the perception and modeling of fine-grained features. Although slight over-activation may occur in some high-frequency non-lesion textures within complex backgrounds, the overall attention remains focused on target regions without introducing significant interference to the detection results.

#### 3.3.2 Position comparison of WDAConv

After confirming the superior performance of WDAConv among various convolutional alternatives, we further explored its structural modeling potential by systematically conducting staged replacement experiments across different hierarchical levels within the HGNetv2 backbone. Four replacement strategies were designed accordingly. The results are reported in **Table 5**. Multiple independent runs were performed for all strategies; only the mean ± standard deviation of All-WDAConv’s AP_50_ and AP_50_:_95_ is reported to highlight its stability, while the others are shown using their averaged results.

**Table 5:** Comparison results in different replacement strategies using WDAConv.

Model	Replaced position	$AP_{50}\left(\%\right)$	$AP_{50:95}\left(\%\right)$	Params(M)	GFLOPs
Base	—	82.2	57.3	5.32	7.01
S1-WDAConv	Stage 1	82.8	57.9	5.30	6.98
S2-WDAConv	Stage 2	82.0	57.8	5.33	7.01
S3-WDAConv	Stage 3	83.3	59.1	5.40	7.04
All-WDAConv	**Stage 1- 4**	**83.7 (± 0.1)**	**61.1 (± 0.1)**	**5.43**	**7.05**

The stage-wise replacement experiments presented in **Table 5** systematically reveal the performance-efficiency trade-offs and progressive impact of introducing the WDAConv at different depths within the HGNetv2 backbone. Specifically, replacing the standard 3×3 convolution in Stage 1 (the shallowest layer) with WDAConv results in a reduction of 0.02 million params and 0.03 GFLOPs, while simultaneously improving AP_50_ and ${\text{AP}}_{50:95}$ by 0.6 percentage points. This indicates that the shallow convolution layers contain notable redundancy, and that WDAConv’s multi-scale detail modeling not only reduces computational cost but also immediately enhances textural sensitivity.

In Stage 2, which targets mid-shallow layers, ${\text{AP}}_{50}$ slightly drops to 82.0%, yet ${\text{AP}}_{50:95}$ still increases by 0.5 percentage points compared to the baseline. This suggests that structural adjustment at this level can enhance localization under higher IoU thresholds, although it may temporarily disrupt semantic alignment between shallow and deep layers.

When extended to Stage 3, which comprises two WDA-Blocks in deeper layers, the model gains substantial improvements in semantic representation: AP_50_ increases to 83.3% and AP_50_:_95_ rises to 59.1%, with only a marginal increase in params of 0.08 M and computation of 0.03 GFLOPs. These results validate the effectiveness of WDAConv in enhancing deep-layer semantic encoding and inter-channel interaction.

Under the All-WDAConv configuration, where Stages 1 to 4 are entirely replaced, the model achieves its best overall performance, reaching 83.7% AP_50_ and 61.1% AP_50_:_95_, marking improvements of 1.5 and 3.8 percentage points over the baseline, respectively, with only modest increases in parameters of about 2% and GFLOPs of about 0.5%.

Overall, the performance evolution exhibits a “fluctuation-to-saturation” trend: shallow-layer replacement primarily reduces redundancy and enhances early-stage texture encoding, mid-layer replacement experiences mild instability due to inter-layer semantic coupling, and deep-layer replacement effectively improves multi-scale semantic contributions to high-IoU accuracy. Ultimately, the unified four-stage replacement achieves an optimal balance between detection accuracy and computational efficiency, without significantly increasing deployment cost.

### 3.4 Ablation experiments of the proposed LE-PWDNet

To evaluate the contribution of each key component in the proposed model to the early-stage detection of PWD, a series of ablation studies were conducted. Specifically, the experiments independently assess the impact of the following modules: WDAConv in the backbone network, ConvFFN in the hybrid encoder, CGAFusion in the feature pyramid, and DySample-E in the FPN. The baseline model used for comparison is the lightweight DEIM-D-Fine-N, which excludes all of the aforementioned components. The detailed ablation results are summarized in **Table 6**. Multiple independent runs were conducted for all ablation settings; to highlight the stability of the full configuration, **Table 6** reports only the mean ± standard deviation of the final LE-PWDNet’s AP_50_, while the other variants are shown using their averaged results.

**Table 6:** Ablation experiment results.

Model	${\text{AP}}_{50}\left(\%\right)$					Params(M)	GFLOPS	FPS
	PWD-E	PWD-M	PWD-L	PWD-D	All			
DEIM(Baseline)	76.9	86.2	87.5	82.6	83.3	5.32	7.01	120
Baseline + A	77.6	86.8	88.5	83.8	84.2	5.43	7.05	120
Baseline + B	77.8	87.1	88.4	83.7	84.3	5.47	7.02	122
Baseline + C	77.1	86.6	87.9	83.0	83.7	5.37	7.03	118
Baseline + D	78.6	87.5	89.1	84.1	84.8	5.33	6.79	126
Baseline + A+B	78.9	87.9	89.4	85.7	85.5	5.56	6.83	127
Baseline + A+B+C	79.4	88.7	89.9	86.1	86.0	5.62	7.08	121
LE-PWDNet	**83.8 (± 0.15)**	**92.4 (± 0.1)**	**94.0 (± 0.1)**	**90.5 (± 0.2)**	**90.2 (± 0.14)**	**5.64**	**7.08**	**125**

The abbreviations denote the following modules: A represents the WDAConv, B represents the ConvFFN module, C represents the CGAFusion module, and D represents the DySample-E module.

**Table 6** indicates that the four modules form a progressive loop of detail enhancement, high-frequency compensation, semantic alignment, and edge reconstruction, yielding a nonlinear synergistic effect. WDAConv strengthens high-frequency and edge information with a very small cost of about 0.11 M parameters and 0.04 GFLOPs, raising overall AP_50_ from 83.3% to 84.2% while maintaining FPS at 120, thereby supplying stable high-frequency and edge representations that support higher-level semantic modeling without compromising inference speed. ConvFFN alone offers moderate gains with AP_50_ reaching 84.3%, accompanied by a slight increase in FPS to 122. However, in combination with WDAConv, it becomes an inflection point for both efficiency and accuracy, lifting overall AP_50_ to 85.5% while reducing GFLOPs to 6.83 and simultaneously boosting FPS to 127, thus improving both accuracy and inference efficiency. Building on this, CGAFusion performs content-guided cross-layer alignment that further stabilizes semantic fusion under complex backgrounds, increasing overall AP_50_ to 86.0%, though FPS slightly decreases to 121 due to added semantic modeling overhead. Finally, introducing DySample-E with edge-guided dynamic upsampling amplifies the synergy, elevating overall AP_50_ to 90.2% while keeping resource usage nearly identical to the baseline with 5.64 M parameters and 7.08 GFLOPs and preserving high inference speed at 125 FPS.

Stage-wise results show the largest benefits at the early and dead stages, with PWD-E reaching 83.8% and PWD-D reaching 90.5%, corresponding to increases of about 6.9 and 7.9 percentage points over the baseline. Experimental results demonstrate that the synergistic mechanism of detail enhancement, high-frequency compensation, semantic alignment, and edge reconstruction effectively strengthens discriminative power for early-stage detection while preserving a sound balance between accuracy, computational efficiency, and real-time performance, thereby offering a practical and deployable pathway for forestry disease detection in real-world settings.

### 3.5 Detection analysis in different scenarios

**Figure 15:**
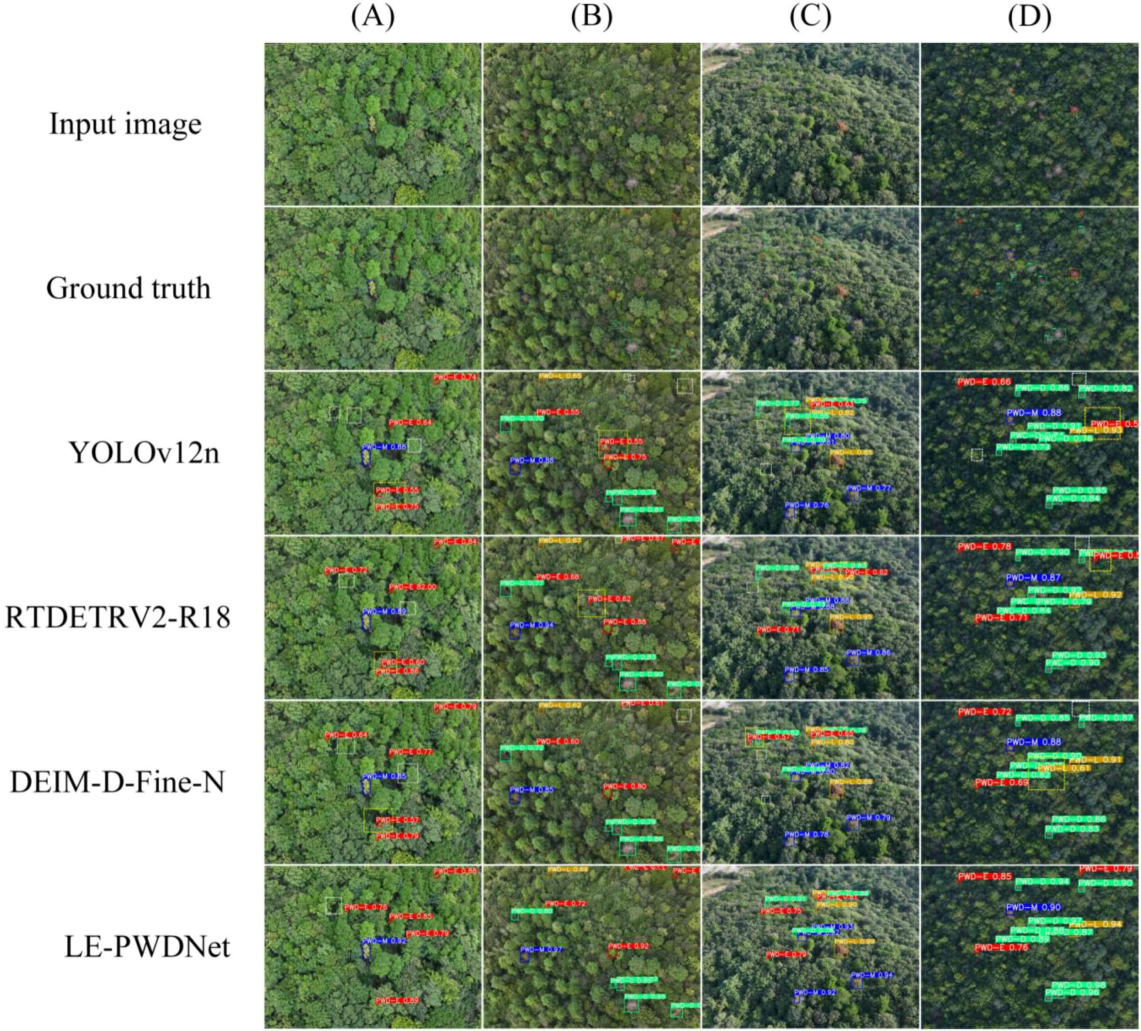
Detection results of different models on typical PWD scenarios. **(A)** Early-stage targets with fine-grained lesions under complex forest background. **(B)** Broadleaf interference scene prone to false positives. **(C)** Oblique-view scene with strong canopy occlusion. **(D)** High-altitude UAV imagery with dense and small targets.

To intuitively demonstrate the performance differences among detection models in the PWD identification task, four representative images were selected from the constructed PWD dataset. These images correspond to typical infection stages, complex background scenarios, and diverse imaging conditions. A systematic visual comparison was conducted on four representative models: LE-PWDNet (Ours), DEIM-D-Fine-N, RT-DETRv2-R18, and YOLOv12n, as illustrated in **Figure 15**. The selected samples comprehensively cover the four key pathological stages of PWD (PWD-E, PWD-M, PWD-L, PWD-D), while also considering challenging environmental factors such as dense forest regions, mountainous terrain, interference from broadleaf trees, and canopy occlusion. This setup ensures a broad and targeted validation of model robustness and generalization ability.

Specifically, **Figure 15A** focuses on early-stage infected targets characterized by subtle lesions and high spatial density, posing significant detection challenges. **Figure 15B** contains multiple broadleaf wilt regions that are visually similar to PWD symptoms, increasing the risk of false positives. **Figure 15C** features an oblique imaging view with substantial canopy occlusion. **Figure 15D** captures high-altitude UAV imagery with dense and small-sized targets, prone to missed detections. Through this visual comparison across four challenging real-world scenarios, the study aims to comprehensively evaluate the capabilities of different models under complex conditions, thereby highlighting the advantages of LE-PWDNet in early-stage recognition accuracy, small object detection, and robustness against background interference.

The visual comparison results (evaluated at a confidence threshold of 0.5) reveal that LE-PWDNet demonstrates superior performance across all four representative scenarios, with only one instance of missed detection (FN = 1) and zero false positives (FP = 0). In contrast, the remaining models show varying degrees of performance degradation: RT-DETR (FN = 4, FP = 4), DEIM-D-Fine-N (FN = 7, FP = 3), and YOLOv12-n (FN = 10, FP = 4).

In **Figure 15A**, which focuses on early-stage subtle lesions, LE-PWDNet successfully identifies low-contrast PWD-E targets with a confidence range of 0.76–0.89, missing only one target. By comparison, DEIM and YOLOv12-n missed 2 and 3 targets, respectively, indicating insufficient feature expressiveness. **Figure 15B** features broadleaf tree interference; only LE-PWDNet achieves complete detection without any false positives, while all other models incur false detections, with YOLOv12-n missing up to three targets.

**Figure 15C** evaluates detection under occlusion and oblique perspectives. LE-PWDNet achieves full recall with zero false positives, whereas the other models suffer from varying degrees of FN and FP, highlighting LE-PWDNet’s robustness to viewpoint variation and scale changes. **Figure 15D** addresses high-altitude dense target detection; LE-PWDNet again achieves zero FN and FP, demonstrating strong capability in small-object recovery and feature aggregation. By contrast, lightweight models such as DEIM and YOLOv12-n perform noticeably worse under these challenging conditions.

Overall, LE-PWDNet, with only 5.64M parameters and 7.09 GFLOPs, is optimized specifically for early-stage PWD-E detection under resource-constrained settings. It consistently outperforms mainstream baseline models across diverse, complex scenarios, including subtle lesions, complex backgrounds, occlusion, and high-density targets. In particular, LE-PWDNet maintains stable detection performance for low-contrast early-stage disease symptoms while achieving an extremely low false positive rate and strong detection robustness.

In conjunction with the quantitative results, LE-PWDNet achieves AP_50_ gains of 4.7, 6.9, and 8.7 percentage points over RT-DETR, DEIM-D-Fine-N, and YOLOv12-n respectively in the PWD-E stage, along with an overall AP_50_ improvement of 4.8%. These findings indicate that LE-PWDNet significantly enhances the accurate identification of early-stage lesions while maintaining high computational efficiency, aligning well with the urgent real-world need for early detection, rapid response, and precise prevention of PWD.

### 3.6 Cross-regional generalization evaluation

**Figure 16:**
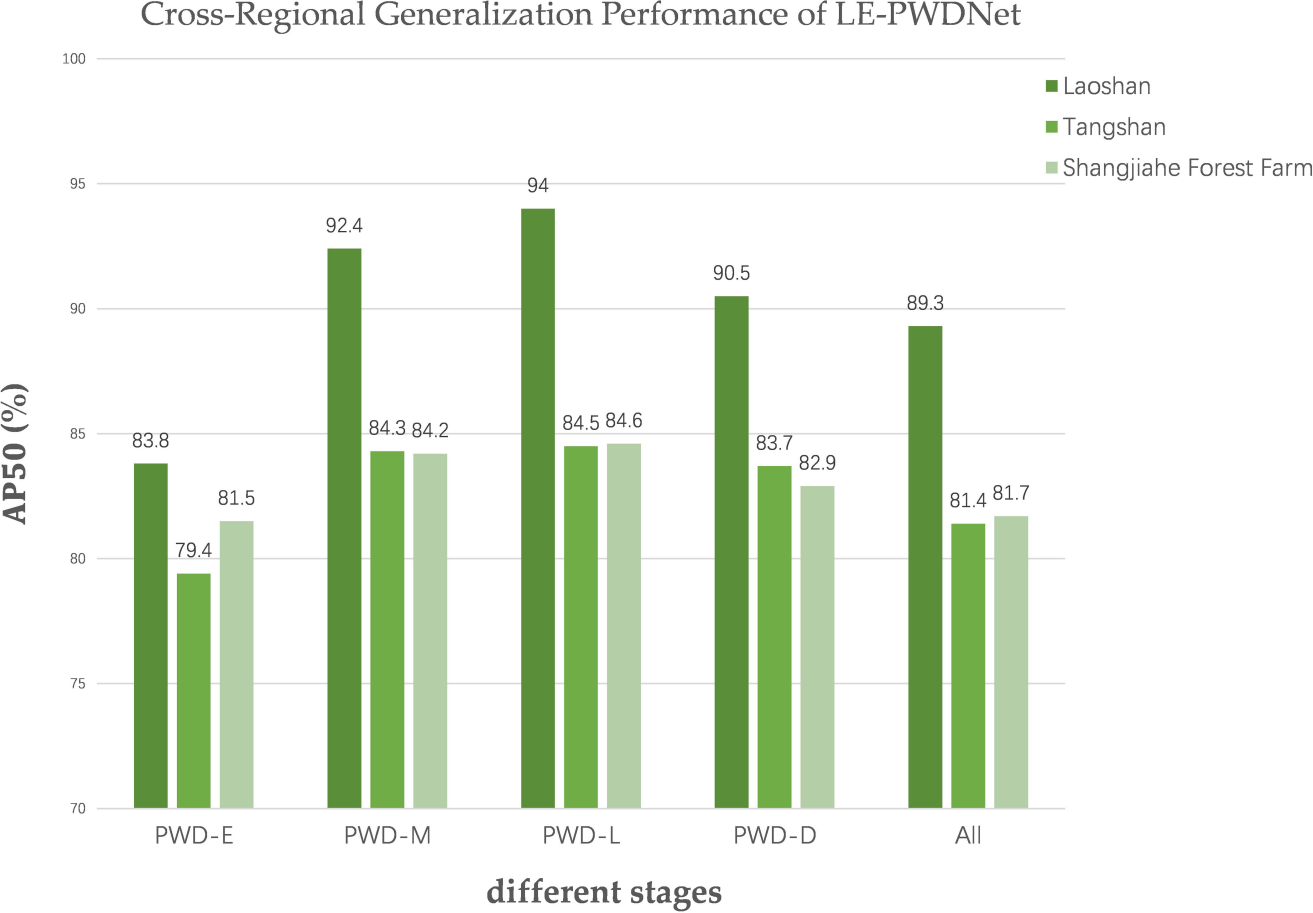
tCross-Regional Generalization Performance of LE-PWDNet.

Although in-domain evaluation on the Laoshan dataset can reflect the model’s performance in its native ecological environment, it remains insufficient to fully verify its adaptability under diverse ecological conditions. To address this limitation, under strict zero-shot conditions, the LE-PWDNet model that was trained solely on the Laoshan dataset comprising 36,845 images across four infection stages was directly applied to two independent datasets from the Tangshan Scenic Area in Jiangning District, Nanjing, and the Shangjiahe Forest Area in Fushun City. The two datasets comprise 2,560 and 3920 high-resolution raw images and differ markedly from Laoshan in terrain, forest structure, dominant tree species, and lighting conditions, thereby providing more challenging test environments for cross-regional generalization assessment. The evaluation employed the same metrics as the in-domain experiments, and performance comparison plots were generated to quantify accuracy variations across different infection stages and to analyze feature transfer behavior (**Figure 16**). The results indicate that LE-PWDNet maintained high detection accuracy for early-stage infection under zero-shot transfer to Tangshan and Shangjiahe, with overall cross-regional performance changes remaining moderate, thus demonstrating strong generalization stability.

**Figure 17:**
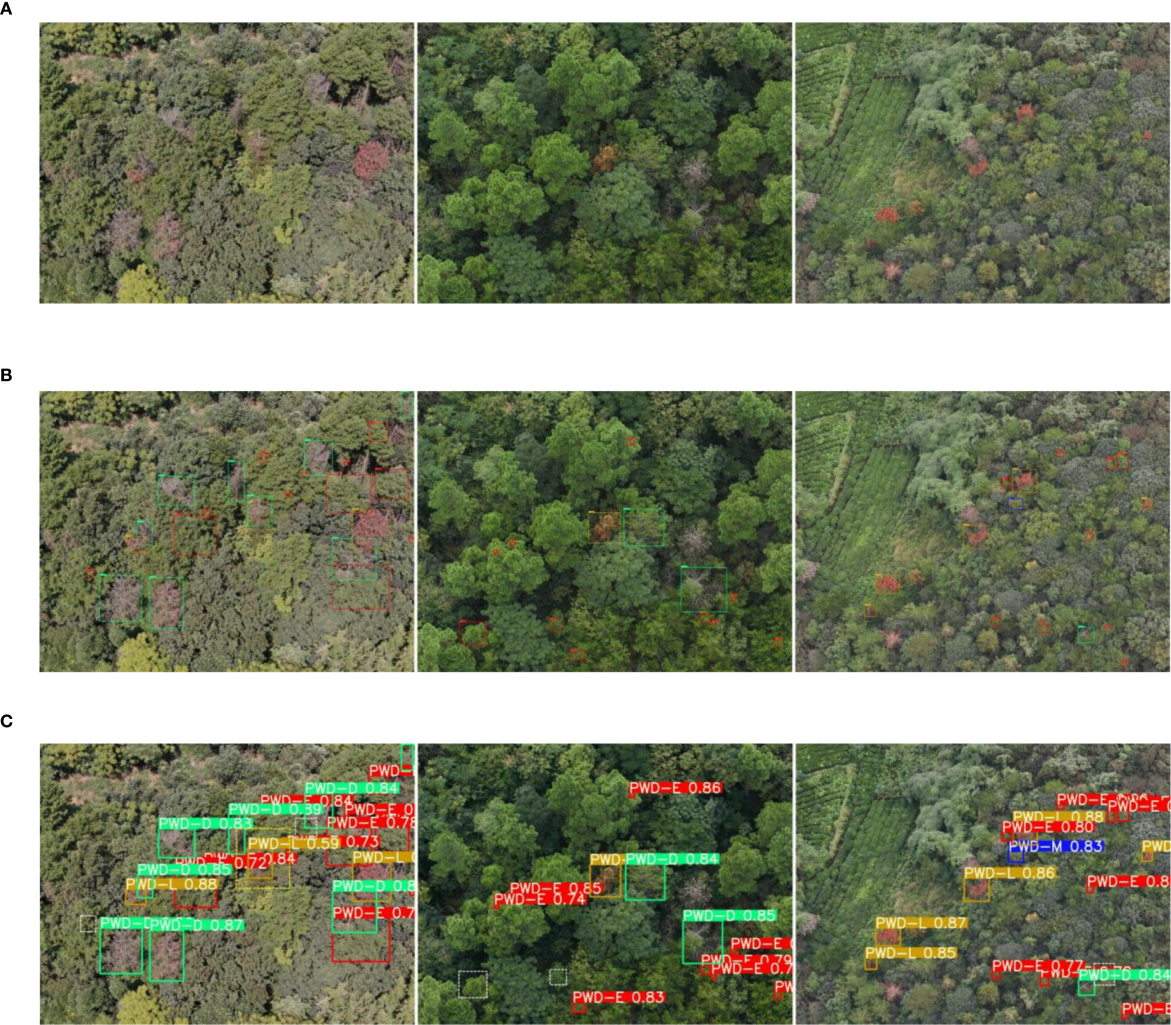
Detection Examples from the Tangshan Dataset. **(A)** Input image. **(B)** Ground truth. **(C)** Result.

**Figure 18:**
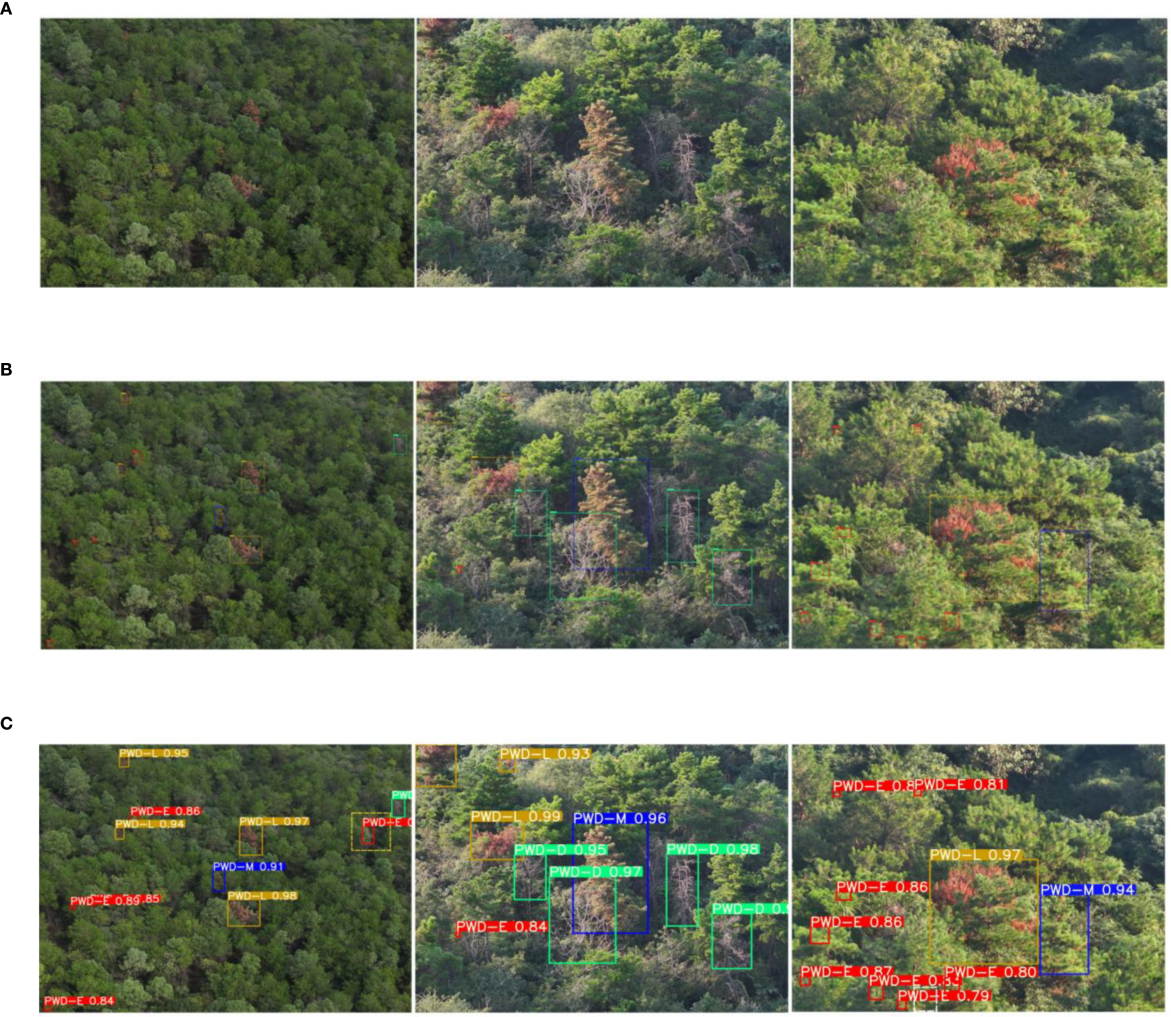
Detection Examples from the Shangjiahe Dataset. **(A)** Input image. **(B)** Ground truth. **(C)** Result.

Furthermore, **Figures 17**, **18** present representative detection visualizations from the two datasets. These cases were captured in forest environment with insufficient illumination and contain densely distributed early-stage infection instances with subtle symptoms, along with interference from other tree species and substantial foliage occlusion. Under such complex conditions, the model was still able to detect the majority of early-stage targets with stable performance; however, five misses and one false detection occurred in the Tangshan dataset, and one miss and one false detection occurred in the Shangjiahe dataset due to lighting and occlusion effects. This observation suggests that under extreme background interference and reduced target visibility, the local texture representation of early symptoms still presents certain challenges. Nonetheless, the overall detection framework maintained high target coverage while keeping the false detection rate at a low level, verifying its robustness in multi-interference scenarios.

Additionally, **Table 7** provides a systematic statistical analysis of detection results on the two datasets. The Tangshan dataset contains a total of 4,051 early-stage infection labels, of which LE-PWDNet successfully detected 3,277 under zero-shot conditions, with 534 false detections and 774 misses. The Shangjiahe dataset contains 6,443 early-stage infection labels, among which 5,886 were correctly detected, with 521 false detections and 648 misses. Overall, the model exhibited strong detection capability in complex cross-regional scenarios, with most false detections concentrated on healthy pine trees or other broadleaf tree targets sharing similar texture patterns with early-stage symptoms. Misses primarily occurred in instances with poor lighting, extremely subtle symptoms, or severe occlusion by other vegetation, indicating that under such conditions, feature signals could benefit from more refined local feature enhancement strategies. This statistical analysis not only reveals the model’s detection characteristics under different interference factors but also provides targeted insights for improving future cross-regional detection performance.

**Table 7:** Evaluation results of LE-PWDNet on independent external datasets.

Independent dataset	TP	FP	FN
Tangshan	3277	534	774
Shangjiahe	5886	521	648

## 4 Discussion

The lightweight detection model proposed in this study, LE-PWDNet achieved a significant performance improvement in PWD detection with only a marginal increase of 0.32M parameters and 0.071 GFLOPs in computational cost, and it elevated the early-stage (PWD-E) precision from 76.9% to 83.8% as well as the overall AP_50_ from 83.3% to 90.2%. This result demonstrates that targeted architectural optimization is more effective than blindly increasing model capacity, particularly for early and subtle lesion detection in resource-constrained environments. The performance gain stems from the synergy between the DEIM training paradigm and modular architectural design. DEIM enhances positive sample density through dense one-to-one matching and introduces a matchability-aware loss to mitigate the effects of low-quality supervision, thereby accelerating convergence and improving early lesion sensitivity. Structurally, the WDAConv leverages wavelet decomposition and detail enhancement to capture high-frequency edge and texture features of early infections, while the ConvFFN module embeds a 5×5 depthwise separable convolution into the feed-forward path to compensate for the low-pass filtering limitations of Transformers. CGAFusion and DySample-E further reinforce cross-layer semantic alignment and spatial reconstruction, effectively reducing missed detections and raising the overall AP_50_ to 90.2%. Notably, approximately 78% of the total performance gain originates from the PWD-E stage, highlighting the critical role of high-frequency detail modeling in early detection. Under a computational load of around 7 GFLOPs, LE-PWDNet achieves an excellent balance between accuracy and efficiency compared with other lightweight detectors. For instance, YOLOv12n, though compact with 2.51M params and 5.82 GFLOPs, attains only 75.1% precision in PWD-E detection, revealing its limitations in capturing weak lesion features. In contrast, LE-PWDNet delivers higher accuracy without sacrificing efficiency, making it well-suited for time-sensitive forestry applications. With 5.64M parameters and approximately 7 GFLOPs, the model supports real-time deployment on Raspberry Pi embedded platforms and can be integrated into UAV systems for forest monitoring and rapid disease alerting.

**Figure 19:**
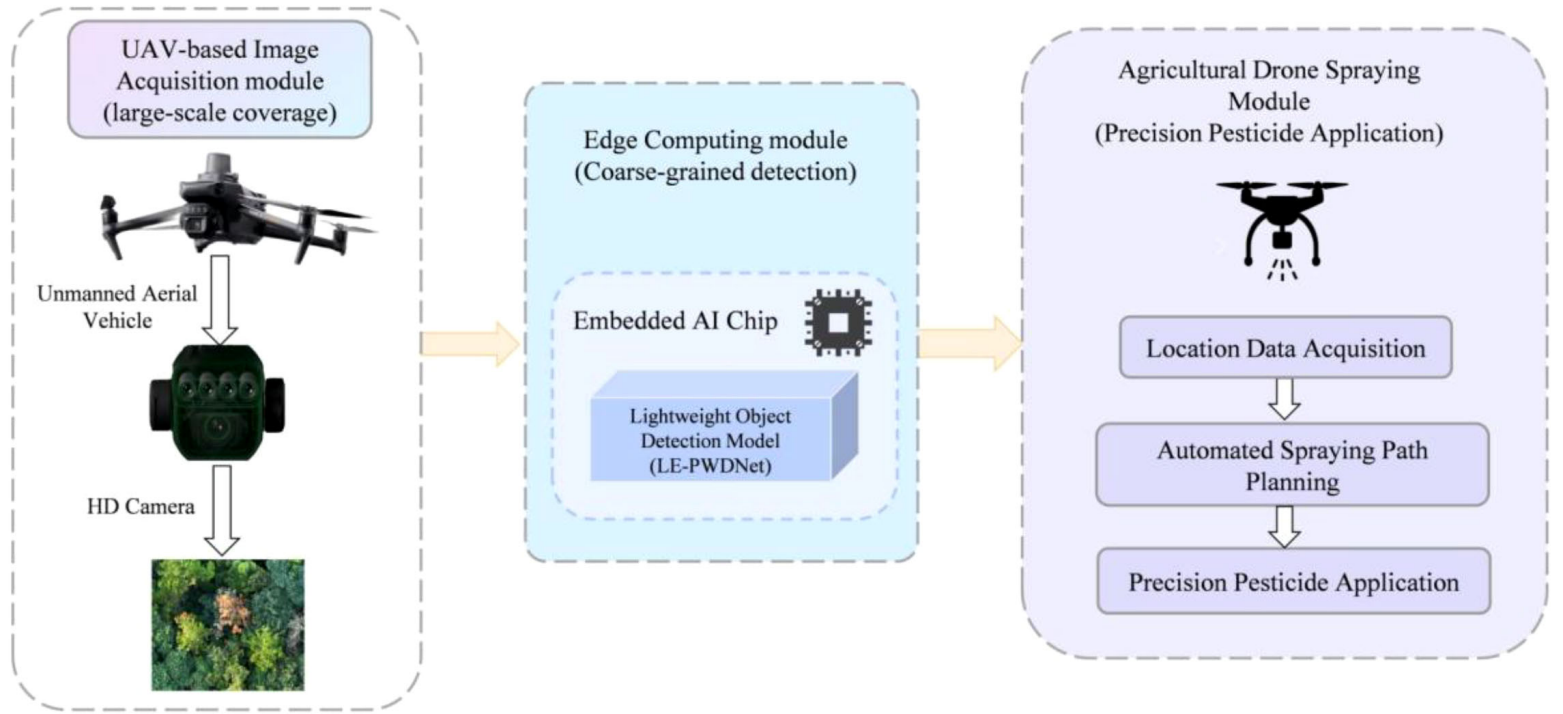
UAV-based Closed-loop System for Early Disease Detection and Precision Pesticide Application.

However, accuracy gains achieved solely through model-architecture and training-strategy optimization remain insufficient for rapid and effective disease control in forestry production. To address this limitation, future work will operationalize LE-PWDNet as an executable, closed-loop forest health management system aligned with **Figure 19**. The pipeline integrates a UAV-based image-acquisition module that enables large-scale coverage, an edge-computing module equipped with an embedded AI chip that executes the lightweight LE-PWDNet for coarse-grained real-time detection, and an agricultural-drone spraying module designed for precision pesticide application. During flight, the edge module extracts GPS-referenced positions of infected-tree candidates and transfers them to the spraying unit, which then automatically generates optimized spraying paths while accounting for no-fly zones, terrain slope, wind speed, and buffer zones, ultimately dispatching drones for targeted application. Furthermore, AI-based path-optimization algorithms, including coverage path planning and reinforcement-learning-based route refinement, will be investigated to minimize pesticide usage and flight time while maximizing coverage and spraying efficiency without compromising operational safety. Edge deployment reduces latency, decreases bandwidth requirements, and ensures autonomy under unstable communication links, thereby enhancing robustness in complex forest environments. Importantly, the proposed system is not limited to PWD but can be extended to other susceptible conifer species, such as Picea and Abies, which are widely distributed in mixed forests. By leveraging LE-PWDNet’s strengths in high-frequency detail modeling and semantic alignment, the framework enables cross-species transfer and domain generalization without necessitating extensive new data collection and annotation, thus offering a practical and ecologically meaningful solution for large-scale, multi-species forest health monitoring and precision management.

## 5 Conclusions

This study presents LE-PWDNet, a lightweight yet highly effective detection model tailored for early-stage PWD identification. By focusing on architectural refinement and training paradigm innovation rather than conventional parameter stacking, the proposed model achieves a remarkable balance between accuracy and computational efficiency. The results underscore the feasibility of deploying LE-PWDNet on resource-constrained platforms, offering a practical solution for real-time, UAV-based forest disease monitoring. The model’s strength lies not only in its detection accuracy but also in its ability to capture subtle lesion features under challenging conditions, which is an essential capability for early intervention and effective containment. Beyond model performance, this work provides a scalable foundation for intelligent forest health management. Future integration with weakly supervised learning, multi-modal sensing, and autonomous UAV systems is expected to further enhance the model’s adaptability and ecological impact. Overall, LE-PWDNet advances both the technical frontier and the practical implementation of precision forestry disease detection.

## Data Availability

The datasets presented in this article are not readily available due to privacy restrictions but can be obtained from the corresponding author upon reasonable request. Requests to access the datasets should be directed to HL, haifeng.lin@njfu.edu.cn.
