# Supplementary figures and images for: LE-PWDNet: a lightweight and enhanced detection framework based on DEIM for early-stage pine wilt disease
Source: Front Plant Sci. 2025 Dec 15;16:1701009. doi: 10.3389/fpls.2025.1701009 (PMC12750628; doi:10.3389/fpls.2025.1701009)

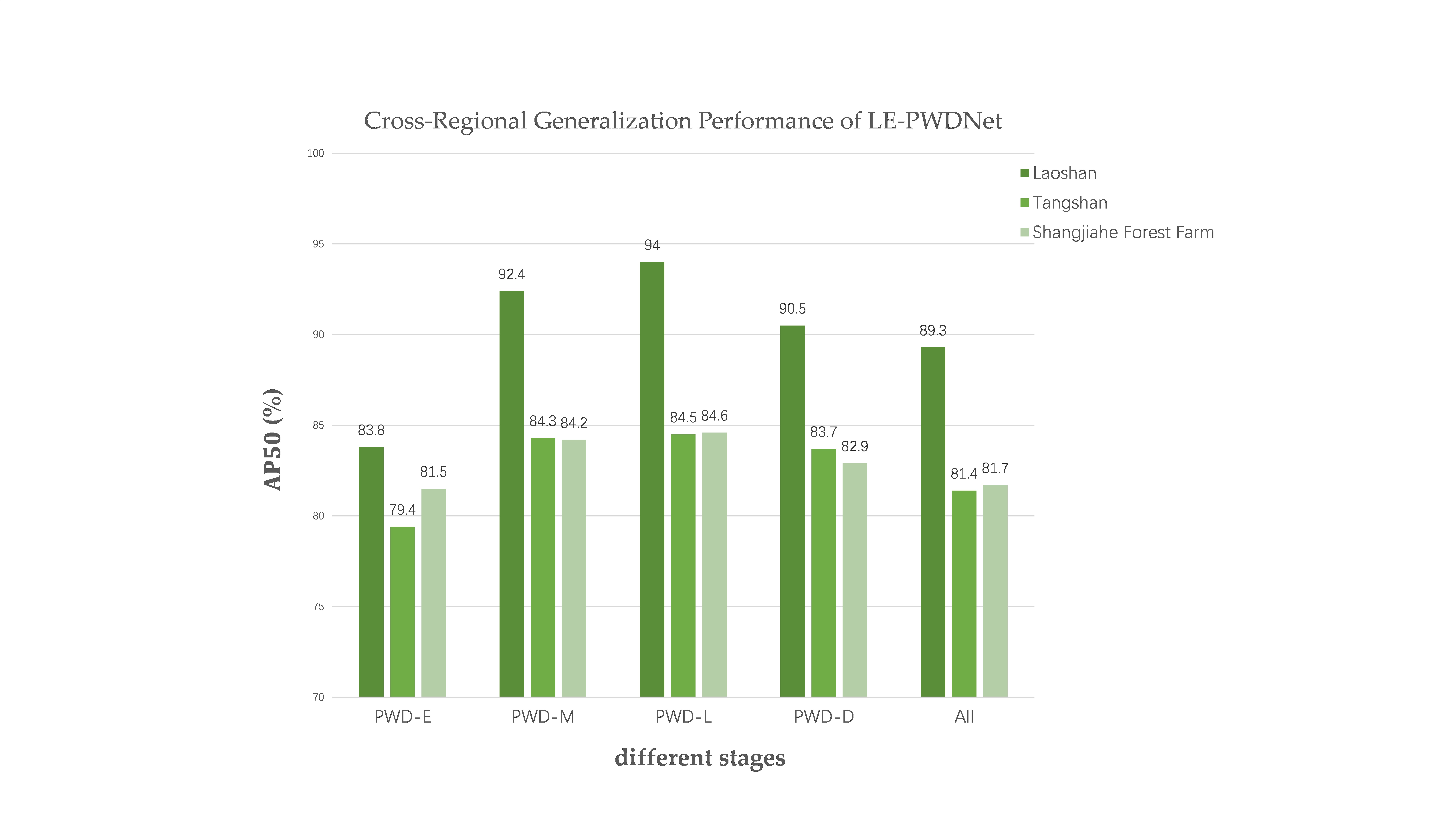

Supplement: Supplementary file 1 [file Image1.jpg]

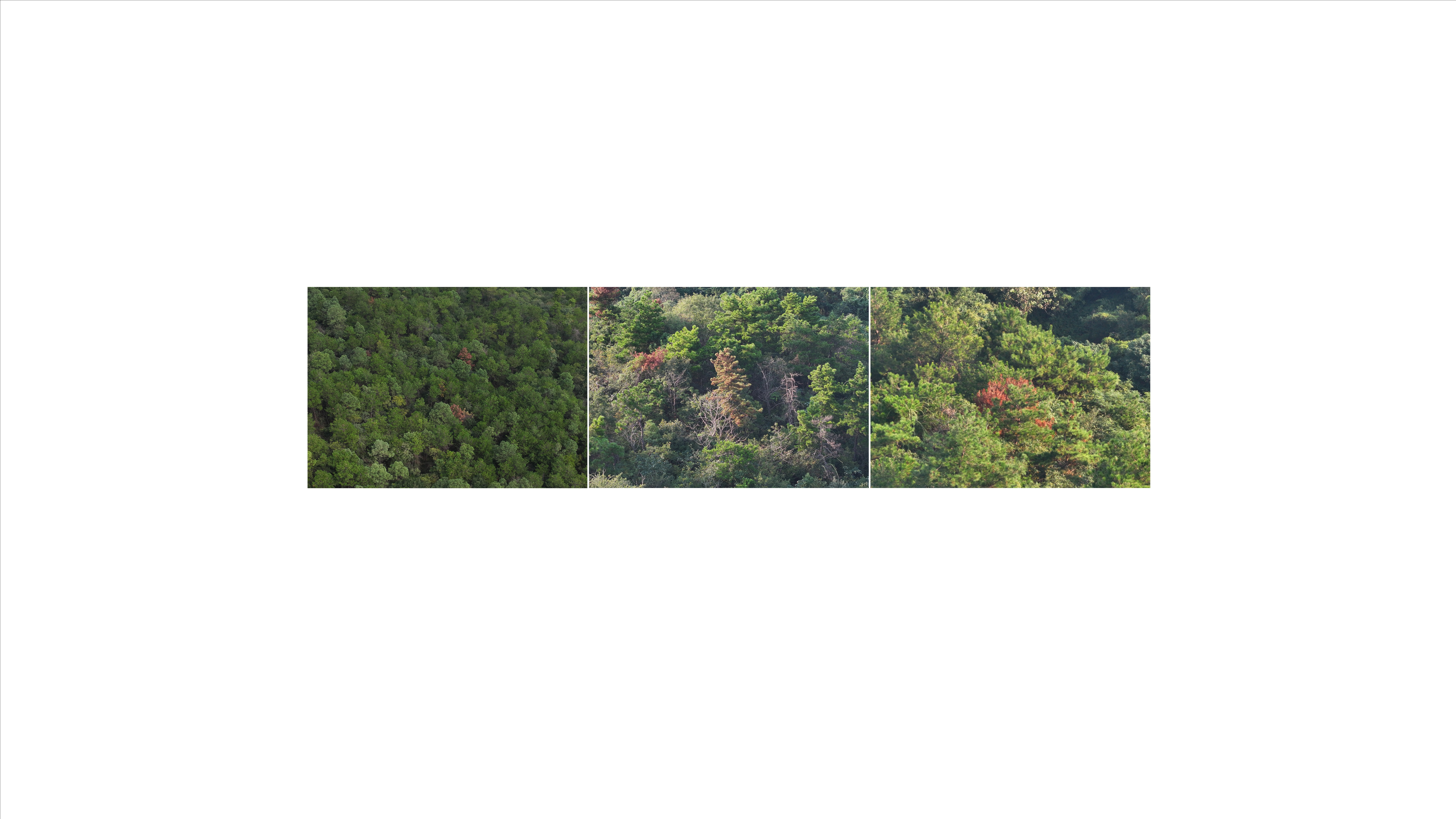

Supplement: Supplementary file 3 [file Image3.jpeg]

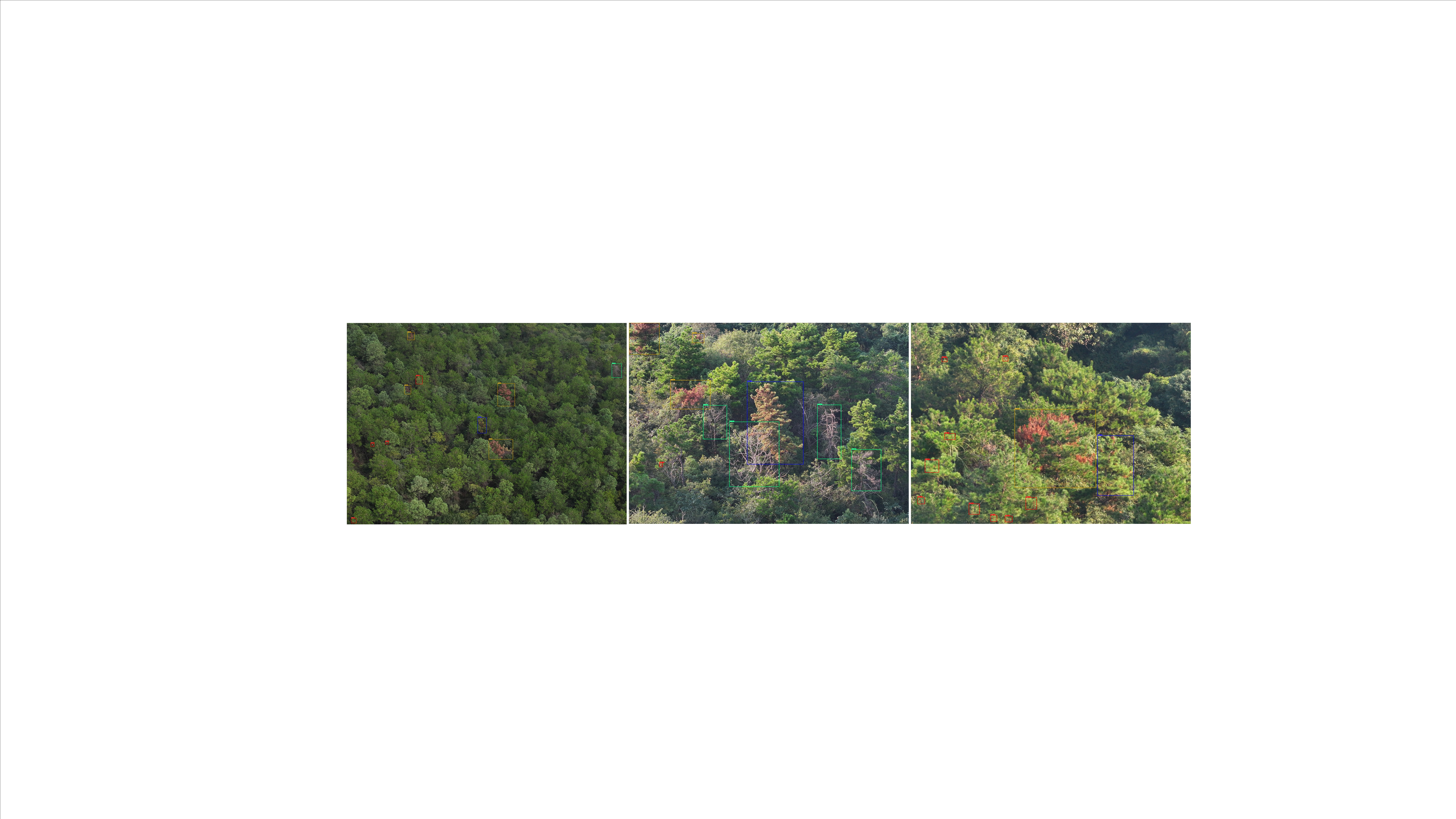

Supplement: Supplementary file 4 [file Image4.jpeg]

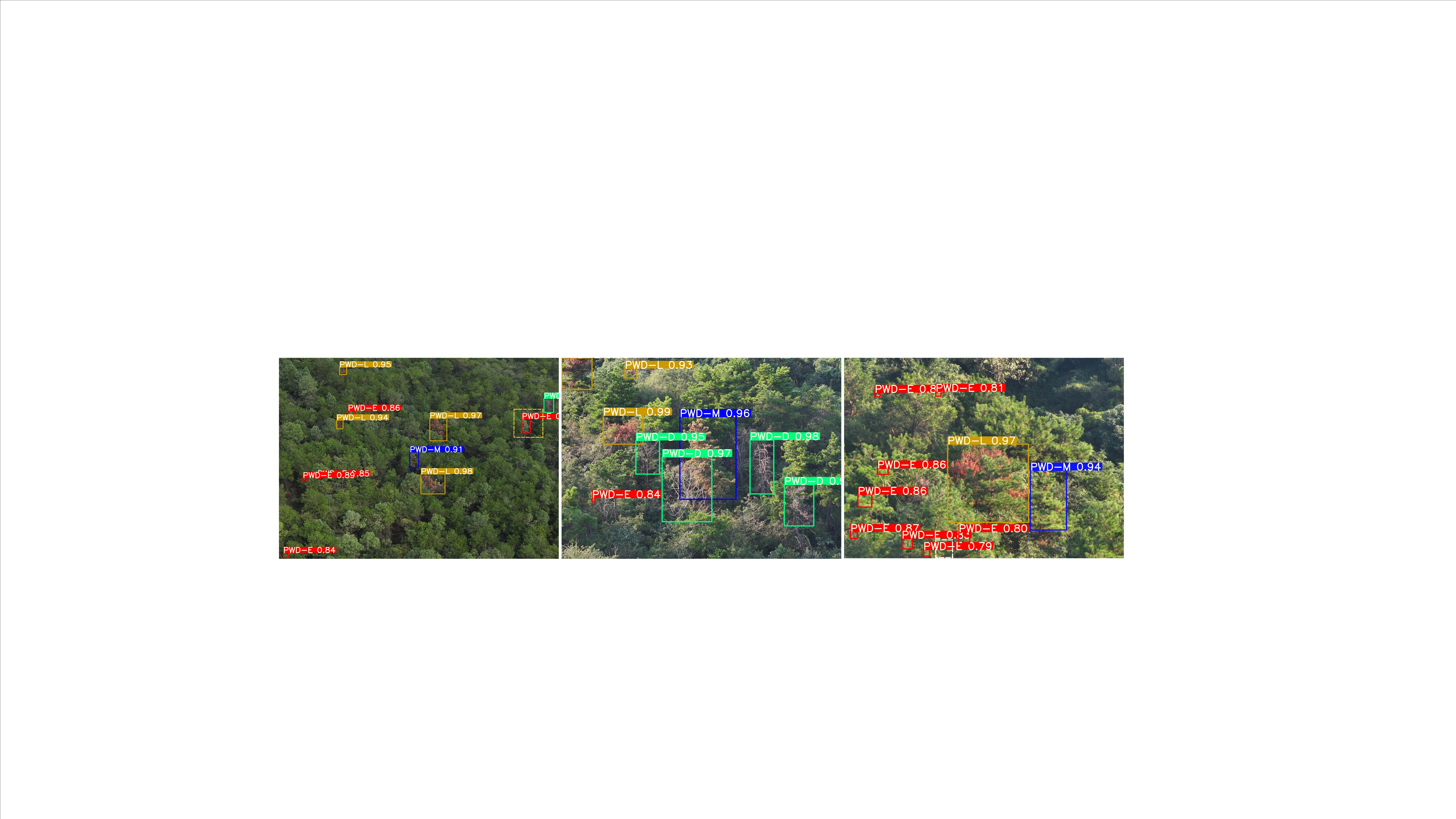

Supplement: Supplementary file 5 [file Image5.jpeg]
